# School‐based health and nutrition interventions addressing double burden of malnutrition and educational outcomes of adolescents in low‐ and middle‐income countries: A systematic review

**DOI:** 10.1111/mcn.13437

**Published:** 2023-03-30

**Authors:** Sachin Shinde, Dongqing Wang, Gretchen E. Moulton, Wafaie W. Fawzi

**Affiliations:** ^1^ Department of Global Health and Population Harvard T.H. Chan School of Public Health Boston Massachusetts USA; ^2^ Center for Inquiry into Mental Health Pune India; ^3^ Department of Epidemiology Harvard T.H. Chan School of Public Health Boston Massachusetts USA; ^4^ Department of Nutrition Harvard T.H. Chan School of Public Healt Boston Massachusetts USA

**Keywords:** adolescents, effectiveness, low‐ and middle‐income countries, nutrition, diet and education outcomes, school health and nutrition programmes, systematic review

## Abstract

School system is a promising platform for addressing all forms of malnutrition in adolescents. However, little is known about the impact of integrated school health and nutrition programmes on adolescent nutrition and educational outcomes in low‐ and middle‐income countries (LMICs). This systematic review sought to characterize school‐based health and nutrition interventions among adolescents in LMICs and analyze their effects on nutritional status and educational outcomes. Four databases were searched for studies evaluating school‐based health and nutrition interventions for adolescents in LMICs, reporting changes in either nutritional status or educational outcomes. A narrative synthesis was used to analyze and describe the evidence. Our review included 68 articles evaluating 58 interventions, of which a third had moderate to strong methodological quality. Forty‐two studies evaluated single‐domain interventions, while 26 evaluated multi‐component interventions. A third of all interventions were based on a theoretical framework. Three‐fourths of the interventions were shorter than 11 months, which may make identifying their effect difficult. The results of the effectiveness of these interventions were mixed and inconsistent across intervention types. Sixteen out of 21 studies evaluating multi‐component interventions and 12 out of 23 studies evaluating nutrition education reported improving nutritional or diet‐related outcomes. One out of six studies reported positive effects on educational outcomes. Our review has identified that research needs include: a greater inclusion of theory‐based approaches to guide the implementation of interventions; more studies of integrated interventions that involve parents and the wider community in LMICs; and extension of outcomes beyond nutritional status to include educational outcomes.

## Key messages

- Developing countries are experiencing nutritional and epidemiological transitions in their adolescent populations that result in widespread undernutrition and increasing overweight and obesity, creating a ‘double burden of malnutrition’.
- The school system offers a promising opportunity to address all forms of malnutrition in adolescents. It is still unclear, however, how school‐based health and nutrition interventions affect adolescents in low‐ and middle‐income countries (LMICs).
- Our review highlights the need for school‐based integrated multi‐component interventions that are contextually appropriate to address all forms of malnutrition and education outcomes in adolescents in LMICs.

## 1 INTRODUCTION

About 1.2 billion adolescents make up roughly 16% of the world's population today, with 90% living in low‐ and middle‐income countries (LMICs) (UNFPA, 2019). Underweight among adolescents is more prevalent than overweight and obesity, although the latter outcomes are increasing in all regions. There has been a reduction in childhood and adolescent underweight, from 37.0% in 2000% to 31.6% in 2016 among boys and from 29.6% in 2000% to 25.9% in 2016 among girls (Development Initiatives, 2020). South Asia is the region with the highest rates of moderate and severe underweight; one in five girls aged 5–19 years and nearly one‐third of their male peers are underweight (Christian & Smith, 2018). Globally, overweight and obesity rates have increased from 13.6% to 27.0% among boys and from 12.8% to 23.1% among girls during the same period. All regions except sub‐Saharan Africa and Asia experienced this trend (Development Initiatives, 2020).

Undernutrition in adolescents results in delayed growth, slowed intellectual development, goitre, increased risk of infection, blindness, anaemia and insufficient mineralization of bone. Obese or overweight adolescents are more likely to experience low self‐esteem, distorted body image, depression, anxiety, discrimination, strained peer relationships and early onset of adult chronic diseases such as type 2 diabetes and hypertension (Bundy et al., 2017). As a result of the current COVID‐19 pandemic, malnutrition in all its forms has been worsening in the LMICs, with economic pressures increasing food insecurity and decreasing access to health care (Zemrani et al., 2021). Furthermore, the closure of schools in developing countries has halted school feeding programmes, an important source of nutrition among millions of school‐goers (Azevedo et al., 2020).

Schools provide an ideal setting for health and nutrition programmes since children spend a large amount of time in school where they could be exposed to supportive environments like school health policies, food programmes, nutrition and physical education and physical activity (Patton et al., 2016). Yet, the evidence from systematic reviews and meta‐analyses is mainly from single‐domain interventions such as nutrition education, physical activity or micronutrient supplements that fail to tackle the multiple drivers of malnutrition (WHO, 2021). Growing evidence has emerged on integrated multi‐component interventions for addressing multiple forms of malnutrition among school‐aged children in recent years. These multi‐component interventions include but are not limited to nutrition and physical education, marketing and promoting healthier foods and beverages, encouraging participation in school meal and snack programmes, role‐modelling healthy eating behaviours and physical activity, and providing free drinking water and sanitation and hygiene services to students inside and outside of school. However, the evidence on these interventions primarily comes from high‐income countries and hence has limited generalizability to resource‐limited settings (Jacob et al., 2021; Langford et al., 2015; Meiklejohn et al., 2016; O'Brien et al., 2021; Russ et al., 2015). The purpose of this review was to characterize school‐based health and nutrition interventions for adolescents in LMICs and describe their effects on nutrition and educational outcomes.

## 2 MATERIALS AND METHODS

The protocol for this systematic review has been described elsewhere (Shinde et al., 2021) and follows the Preferred Reporting Items for Systematic Review and Meta‐analysis (PRISMA) guideline (Moher et al., 2015) (Supporting Information: File 1), with PROSPERO registration no. CRD42020211109.

### 2.1 Eligibility criteria

To be eligible for inclusion, studies had to be: randomized controlled trials (RCTs) and controlled before‐after studies (CBAs); conducted in school‐going adolescent boys and girls aged 10−19 years, or the age range of the participants overlapped with this window; primary research that evaluated one or more interventions including nutrition education, physical activity, school gardens, school food and nutrition policies, school environment, and water, sanitation, and hygiene (WASH); reported changes in at least one adolescent outcome related to nutrition, or education; and published and unpublished studies in the English language from LMICs, with no restrictions on the year.

Studies were excluded if CBAs did not account for the baseline differences between study arms; conducted among school‐going adolescents with specific medical conditions; and studies evaluating the impact of educational interventions only on educational outcomes. We also excluded studies examining the impact of only school feeding and micronutrient supplementation or fortification as these were covered by recent reviews in the field (Salam et al., 2016; D. Wang et al., 2021).

### 2.2 Information sources and search strategy

A comprehensive search of MEDLINE (through PubMed), Embase, CENTRAL (through the Cochrane Library), CINAHL and Google Scholar was conducted to identify potentially eligible studies. Key search terms related to population (adolescents, young people, youth, high school, secondary school); interventions (nutrition education, physical activity, school food policy, school environment, school gardens, WASH); types of studies (RCTs, CBAs); and settings (LMICs) were used. The search terms were modified for each database. The specific search strategy for the PubMed database is provided in Supporting Information: File 2.

The reference lists of identified studies and relevant systematic reviews were manually checked for other relevant studies. Records retrieved from electronic databases were imported into Covidence, a streamlined, web‐based software platform for systematic review processes. Titles and abstracts were screened independently. The full‐text screening was conducted in duplicate, as per the inclusion/exclusion criteria. Any disagreements about eligibility were settled through discussion. Study exclusions were documented and summarized using the PRISMA flow diagram (Moher et al., 2015).

### 2.3 Data extraction

Data were extracted using a data sheet that was purposely designed for the study. The following data were extracted: title, first author, journal, year of publication, country, study design, sample size (if a cluster‐randomized controlled trial (CRCT), number of clusters, and average cluster size), sample characteristics (e.g., age and sex of the participants), intervention characteristics (including intervention type, guiding theory/framework, intervention description, duration, delivery mechanisms, and intervention coverage and fidelity), information on comparison intervention, outcomes assessed, time points of assessments and intervention effects. A request for additional information was sent to the corresponding authors of five studies, but no response was received. Despite the lack of complete data, these studies were included in the review due to their relevance.

### 2.4 Risk of bias assessment

Version 2 of the Cochrane risk‐of‐bias tool (RoB‐II), consisting of five domains for RCTs (J. A. C. Sterne et al., 2019) and an additional domain for CRCTs (Elridge et al., 2017), was used to assess the risk of bias. Each domain was rated as ‘low risk of bias’, ‘high risk of bias’, or ‘some concern’. We defined an RCT to be at low risk of bias when the risk of bias was low across all domains; at high risk of bias if there was a high risk of bias in any domain, and having some concerns if there were some concerns in any domain but not of a high risk of bias for any domain.

The Risk of Bias in Non‐randomized Studies of Interventions (ROBINS‐I) tool was used to analyze bias for CBAs (J. A. Sterne et al., 2016). Biases were judged across seven domains where each risk was rated as ‘low bias’, ‘moderate bias’, ‘serious bias’, ‘critical bias’ or ‘no information’. We defined a CBA to be at low risk of bias when the risk of bias was low or moderate across all domains; at high risk of bias if there was a serious or critical risk of bias in any domain and, of some concerns if the assessment had no information for one or more domains but was of low or moderate risk of bias for all other domains.

### 2.5 Quality assessment

The ‘Effective Public Health Practice Project quality assessment tool for quantitative studies’ was used to evaluate each study's methodological quality (Effective Public Health Practice Project, 1998). Six components were considered in rating the studies: selection bias, study design, confounders, blinding, data collection methods, withdrawals and dropouts. Each component was rated as weak, moderate or strong and summed to arrive at an overall score for each study. The study was rated strong if none of the components received weak ratings. Studies with one weak rating were categorized as moderate, while those with two or more weak ratings were categorized as weak.

### 2.6 Data synthesis

We presented the systematic synthesis of all included studies in the text as well as in a table, using the Synthesis Without Meta‐analysis (SWiM) guideline (Campbell et al., 2020). For studies that included more than one follow‐up point, only the results at the last follow‐up point were considered. Intervention effects were summarized as mean differences, Cohen's *d*, or adjusted beta‐estimates for continuous variables and odds ratios or relative risks for binary outcomes. The summary vote‐counting was presented according to the direction of the effect for each outcome (Campbell et al., 2020). Based on the type of intervention and outcome measures, the results were grouped and presented. We could not perform meta‐analyses due to the heterogeneity of interventions and outcomes.

## 3 RESULTS

### 3.1 Description of studies

**Figure 1:**
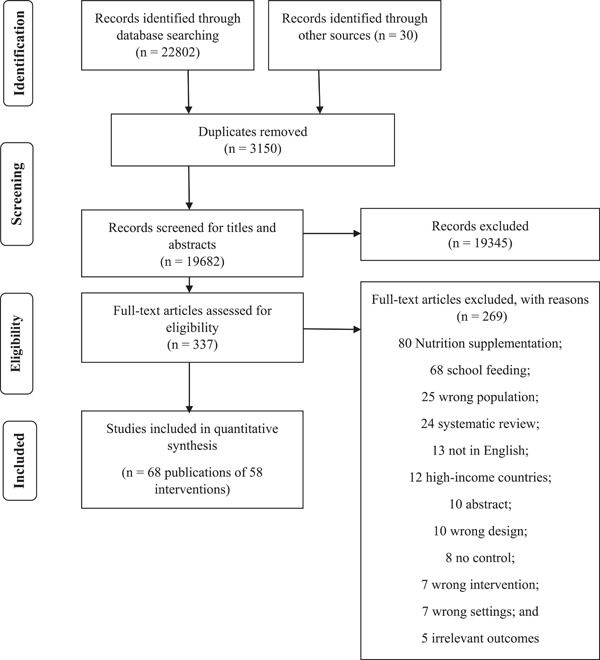
Flow chart for the literature search for intervention studies

Figure 1 shows how references identified through searches were processed for this review. Our searches yielded 19,682 records after the removal of duplicates. Of these, 19,345 were excluded at the title and abstract screening. We reviewed 337 full‐text articles for eligibility. We identified 121 articles that initially appeared to be of relevance to this review but were subsequently excluded for a variety of reasons. These articles included the wrong age group; were not in the English language; were conference or supplement abstracts; did not fulfil the criteria for intervention or outcomes; were from high‐income countries; were not randomized or did not include control. Thus, 68 articles describing 58 interventions met the eligibility criteria for inclusion in the review. Table 1 summarizes study characteristics including study type, study population, sample size, intervention type and duration, the basis for the intervention, comparison intervention, outcomes and overall evidence quality.

**Table 1:** Summary characteristics of the included studies on school‐based health and nutrition interventions targeting multiple forms of malnutrition in adolescents in low‐ and middle‐income countries

First author, year, country	Study type	Target population	Sample	Intervention content	Duration & follow‐up	Theoretical basis	Comparison	Outcomes	Overall quality
*Nutrition education*									
Akdemir et al. (2017), Turkey	CRCT	Boys and girls from Grades 1−8	2 schools; 1288 participants; 53% boys	Lectures on healthy nutrition and active lifestyle, and causes of and preventive strategies for obesity Daily sell of fresh fruits at the school restaurant Two sessions on nutrition with families Distribution of a healthy‐nutrition brochure for families	Duration: 1 school year	None stated	No intervention	Weight status, body mass index (BMI), overweight and obesity and diet quality	Strong
Chagas et al. (2020), Brazil	CRCT	13−16 years old boys and girls	8 schools; 1061 participants; 43% boys	A virtual digital game addressing food classification, healthy eating practices, importance of the act of cooking and food marketing focused on misleading advertisements and importance of reading food labels	Duration: 7−17 days	Social cognitive theory	No intervention	Knowledge of healthy eating, nutritional knowledge, perception of a healthy diet and self‐efficacy	Weak
Cunha et al. (2013), Brazil	CRCT	Boys and girls from Grade 5; mean age 11 years	20 classes; 599 participants; 52% boys	Monthly 1 h long classroom‐based sessions for students, delivered by a nutritionist Use of games, staging of theatre sketches, watching movies and puppet shows, and writing and drawing contests Messages for parents through illustrated booklets on recipes Teachers encouraged to address intervention sessions on healthy eating	Duration: 9 months follow‐up: 6 and 9 months after baseline	Pedagogy of the oppressed	A 1 h orientation on general health and advice on healthy eating	BMI, and consumption of food and beverages	Weak
Brito Beck da Silva et al. (2019), Brazil	CRCT	Boys and girls from Grades 7−9; Mean age 14.5 years	12 schools; 608 participants; 52% boys	16 weekly virtual learning environment sessions using Moodle in the school computer lab Sessions cover learning questions, session feedback, food log, physical activity log, hunger and satiety scale, goals log and discussion forum Printed intervention material for parents and teachers	Duration: 12 months	Cognitive‐behavioural therapy	No intervention	Weight, BMI, waist, and hip circumference, waist‐height ratio and food consumption	Moderate
Fonseca et al. (2019), Brazil	CRCT	Boys and girls from Grade 9	7 schools; 461 participants; 53% boys	Three meetings with students lasting up to 45 min each and within an interval of 7–15 days Topic: Healthy eating and food classification	Duration: 45 days	Ten steps towards healthy eating	No intervention	Dietary knowledge, self‐perceived diet quality and breakfast eating practice	Weak
Karimi‐Shahanjarini et al. (2013), Iran	CRCT	12−15 years old girls	29 classes; 739 participants	Three intervention components: information, motivation and implementation Information through 4.5 h per class sessions Motivation through group discussions using a booklet to open questioning, reflection, summarizing and provoking the students' intention to change Discussion on the role of implementation intention in promoting healthy behaviours	Duration: None stated	Theory of planned behaviour	No intervention	Snacking (healthy and unhealthy) behaviour	Weak
Keshani et al. (2019), Iran	CRCT	13−15 years old boys and girls	16 schools; 311 participants; 51% boys	Four 90‐min long teacher‐facilitated interactive sessions Use of collaborative learning techniques such as buzz group discussions, reciprocal teaching with a Jigsaw design and problem‐based learning through a send‐a problem, and role‐play methods A briefing session for the parents and the school health instructors	Duration: None stated	Health belief model	Routine school educational programme	Knowledge of nutrition, and diet quality index	Weak
Leventhal et al. (2016), India	CRCT	Girls from Grades 7−8	76 schools; 3000 participants	Resilience curriculum: 23 weekly sessions to improve girls' psychosocial resilience, or their ability to bounce back from challenges Health curriculum: 21 weekly sessions (two topics on nutrition and anaemia) to improve girls' knowledge of, attitudes about, and behaviours related to physical health issues Intervention delivery by trained peers	Duration: None stated follow‐up: 3‐time points including baseline (exact time points not mentioned)	None stated	No intervention	Knowledge of health and nutrition	Weak
Lin et al. (2017), Iran	CRCT	13−18 years old boys and girls	48 schools; 1413 participants; 51% boys	20 min long group discussion with adolescents on a healthy diet 30 min long group discussion with parents A single brochure for adolescents and parents post‐group discussion	Duration: 1 month follow‐up: 6 months post‐intervention	Health action process approach	No intervention	Fruit and vegetable intake	Moderate
Najimi and Ghaffari (2013), Iran	CRCT	Boys from Grade 4	2 schools; 138 participants	Four weekly sessions of 60 min each for boys Two sessions of 50 min each for parents and teachers	Duration: 4 weeks	Social cognitive theory	No intervention	Fruit and vegetable intake	Weak
Sichieri et al. (2009, 2012), Brazil	CRCT	9−12 years old boys and girls	47 classes, 1140 participants; 47% boys	Ten 1 hlong sessions of activities Classroom quizzes and quizzes using water versus sugar‐sweetened carbonated beverages Composing a song during three 1 h long sessions	Duration: 7 months follow‐up: 1 year post‐intervention	None stated	Two sessions on health issues and a brochure on healthy diets	Weight, BMI, prevalence of overweight and obesity	Moderate
Toral and Slater (2012), Brazil	CRCT	11−19 years old boys and girls	10 schools; 860 participants; 40% boys	Monthly colourful printed magazines that promoted healthy eating Monthly mailed information newsletters directed towards the participants' stages of change	Duration: 6 months	None stated	No intervention	Fruit and vegetable intake	Weak
D. Wang et al. (2013, 2014), China	CRCT	12−14 years old boys and girls	3 schools; 188 participants; 48% boys	26 once a week 15‐min long nutrition education classes Once a month peer group support session Distribution of a brochure every 3 months Display of posters	Duration: 6 months	None stated	No intervention	Knowledge of nutrition	Weak
D. Wang, Stewart, Chang and Shi (2015), D. Wang, Stewart and Chang (2015), China	CRCT	12−14 years old boys and girls	2 schools; 130 participants; 50% boys	Formation of school nutrition group Monthly school nutrition group meetings One 90‐min long school staff nutrition training session Distribution of informational resources for school staff and parents Weekly in‐class curriculum One 90‐min workshop for parents Monthly text messages to parents	Duration: 6 months	Health promoting schools	No intervention	Dietary consumption	Weak
Yusoff et al. (2012, 2013), Malaysia	CRCT	16−17 years old boys and girls	8 schools; 280 participants; 20% boys	Four weekly 1‐h long lectures and Q&A sessions, one exhibition, four video presentations, and two discussions on iron deficiency anaemia and nutrition Distribution of six brochures and two posters	Duration: 3 months	None stated	Daily supplementation of Iron and folic acid and vitamin C capsules	Haemoglobin, and knowledge of nutrition	Weak
Amani and Soflaei (2006), Iran	CBA	16−18 years old girls	2 schools; 60 participants	Group discussions on dietary sources of iron, iron availability, and the signs and consequences of iron deficiency and anaemia and distribution of pamphlets	Duration: 2 months	None stated	No intervention	Nutrition knowledge score, lifestyle score, food frequency score, haemoglobin and serum ferritin	Weak
Dansa et al. (2019), Ethiopia	CBA	11−19 years old girls	2 schools; 132 participants	11 bi‐monthly nutrition education sessions using lesson‐based education, group discussion, demonstrations of pulse recipe preparation skills, posters and brochures	Duration: 6 months	Health belief model	No intervention	Weight, height, BMI and height *z*‐scores and dietary diversity	Weak
Brito Beck da Silva et al. (2015), Brazil	CBA	10−17 years old boys and girls	2 schools; 833 participants; 43% boys	Eight monthly meetings for students to promote healthy eating and physical activity, each lasting 50 min Workshops for students to reinforce knowledge about healthy eating and physical activity A webpage for adolescents Didactic‐educational materials for parents	Duration: 9 months	None stated	No intervention	BMI, and consumption of food and beverages	Weak
Ghrayeb et al. (2013), Palestine	CBA	16−18 years old boys and girls	4 schools; 236 participants; 49% boys	Five classroom‐based lessons on the nutritional value of whole versus processed foods, and types of nutrients and their sources Use of lesson plans, brochures, handouts, PowerPoint presentations and visuals	Duration: 1 month	None stated	No intervention	Knowledge of nutrition	Weak
Hosseini et al. (2015), Iran	CBA	Boys and girls; mean age 13.9 years	4 schools; 88 participants; 50% boys	Five sessions based on collaborative learning focusing on breakfast consumption (attitude, norms, beliefs, and intention) using focus group discussions, speeches, and questions and answers Discussion moderated by trained peers (students)	Duration: None stated	Theory of reasoned action	Routine school educational programme	Knowledge of and attitude towards breakfast consumption, and subjective norms towards breakfast consumption	Weak
Sharif Ishak et al. (2020), Malaysia	CBA	13−18 years old boys and girls	2 schools; 76 participants; 48% boys	A peer‐led, school‐based health promotion programme Eight topics delivered over eight sessions within 16 weeks, with 2‐week gaps between each session of 60–90 min	Duration: 14 weeks follow‐up: 3 months post‐intervention	None stated	Standard health and physical education class at least once a week	BMI *z*‐score, waist circumference, % body fat, and knowledge, attitude and practice of a healthy lifestyle	Weak
Shen et al. (2020), China	CBA	Boys and girls; mean age 13.1	3 schools; 573 participants; 56% boys	Three health education sessions for students and parents Regular monitoring of students' weight and feedback to parents and students via messages on smartphones	Duration: 3 months	None stated	Usual health curriculum in school	BMI, and BMI *z*‐score	Weak
Taghdisi et al. (2016), Iran	CBA	Boys from Grades 4−6	1 school; 184 participants	Four 45‐min long training sessions for students One 60‐min long training session for teachers and parents	Duration: None stated	Theory of planned behaviour	No intervention	Fruit and vegetable intake	Weak
*Physical activity*									
Andrade et al. (2014) and Ochoa‐Avilés et al. (2017), Ecuador	CRCT	Boys and girls from Grades 8−9	20 schools; 1083 participants; 37% boys	Delivery of educational package organized at the classroom level, covering 13 chapters through two books; each chapter taught twice a week over a 90‐min‐long session Six parental workshops Social events like prep talks, walking trails, and posters display	Duration: 24 months follow‐up: 17 and 28 months	Selected behavioural change techniques	Standard curriculum determined by the Ecuadorian government (80 min of physical education)	BMI *z*‐score, waist circumference, total fat overweight and obesity prevalence, breakfast intake, fruit and vegetable intake, added sugar intake, and unhealthy snacking, unhealthy snacking at school	Strong
Gutiérrez‐Martínez et al. (2018), Colombia	CRCT	Boys and girls; mean age 10.5 years	3 schools; 168 participants; 43% boys	Standardized activities lasting 20 min from the 30 min of the usual recess time, three times a week for 10 weeks 30 physical activity sessions that combined games Daily text messaging to students and parents to promote participation in physical activity	Duration: 10 weeks	Part of *Movam Se Estudantes* of the IDRD of Bogotá	No intervention	BMI *z*‐score, and % fat	Weak
Leong et al. (2015), Malaysia	CRCT	16‐year‐old girls	4 schools; 81 participants	Supervised, 1 h aerobic dance session twice a week for 6 weeks 250 ml of regular fresh cow's milk at breakfast 5 days per week	Duration: 6 weeks	None stated	No intervention	Digital span test	Weak
Telles et al. (2013), India	RCT	8−13 years old boys and girls	98 participants	Intervention 1: 45‐min physical exercise session for 5 days a week Intervention 2: 45‐min yoga session for 5 days a week	Duration: 3 months	None stated	Not applicable	BMI, stroop colour‐word naming taskand	Weak
Guimarães et al. (2017), Brazil	CBA	19‐year‐old or younger boys and girls	2 schools; 71 participants; 25% boys	Two weekly sessions of recreational physical activities Controlled 60 min of physical exercises for a period of 14 weeks including warm‐up (5–10 min), main part (40–50 min), and recovery (5–10 min). Monthly instructional meetings hosted by a nutrition specialist	Duration: 14 weeks	None stated	No intervention	Weight, % fat mass, and % fat‐free mass	Weak
Kargarfard et al. (2012), Iran	CBA	14−16 years old girls	206 participants with mothers and 60 participants without mothers	Twice a week physical activity sessions; each of 90 min for girls and their mothers 10 min of warm‐up, 30 min of aerobic activity and stretching exercises, 20 min of a free group playing, and 10 min of cool down	Duration: 12 weeks	None stated	Same intervention but without mothers	BMI	Weak
Li et al. (2014), China	CBA	7−15 years old boys and girls	4 schools; 921 participants; 53% boys	Three physical education components: (i) physical education improvement; (ii) extracurricular physical activity for overweight/obese students and (iii) family physical activity with parent involvement Three health education lectures for students	Duration: 12 weeks	Social‐ecological model of health promotion	No intervention	BMI, overweight/obesity prevalence, waist circumference and abdominal skinfold	Weak
Zhang et al. (2019), China	CBA	14−16 years old boys and girls	2 cohorts; 460 participants; 40% boys	Two weekly 90‐min long specialized sports training sessions including football, volleyball, badminton, table tennis, tennis and aerobics	Duration: 3 semesters	None stated	Traditional cardiovascular fitness curriculum	Academic achievement in the Chinese language, English language and mathematics	Weak
*Nutrition subsidy*									
Chen et al. (2019), China	CRCT	Boys and girls from Grades 4 and 5	59 schools; 866 participants	Nutrition subsidy to the school with a general policy target of ‘malnutrition reduction’ or with a specific target of ‘anaemia reduction’ School heads informed about the objectives of respective policy	Duration: 6 months	None stated	No intervention	BMI *z*‐scores, incidence of underweight, haemoglobin, the incidence of anaemia and dietary diversity	Moderate
*WASH intervention*									
Caruso et al. (2014), Kenya	CRCT	Boys and girls from Grades 1−8	60 schools; 17, 564 participants; 51% boys	Latrine cleaning and handwashing: Provision of reusable hardware (buckets, brooms, hand brushes, plastic scoop), consumables (bleach, powdered soap), toilet tissue, handwashing materials and sheets for pupils to monitor latrines conditions daily Training of two teachers per school	Duration: 1 school year follow‐up: every two weeks post‐intervention for four times	None stated	No intervention	School absence	Strong
Chard et al. (2019), Laos	CRCT	Boys and girls from Grades 3−5; mean age 10.5 years	100 schools; 3, 545 participants; 51% boys	Infrastructure (hardware) and behaviour change (software) components Hardware: Provision of a school water supply, school sanitation facilities and handwashing facilities Software: Clean drinking water, group handwashing, toilet cleaning and school compound maintenance	Duration: 1 year follow‐up: 7, 13 and 19 months after baseline	None stated	No intervention	School absence, enrolment, dropout and grade progression	Moderate
Freeman et al. (2012), Kenya	CRCT	12−14 years old boys and girls	135 schools; 6036 participants; 52% boys	3‐day long training of teachers on water treatment and hygiene promotion Handwashing and drinking water containers and a one‐time supply of water disinfectant Provision of latrines to the students	Duration: 1 year	None stated	No intervention	School absence	Strong
Theriault et al. (2014), Peru	CRCT	9−12 years old boys and girls	18 schools; 1034 participants; 51% boys	Enhanced health hygiene education consisting of booklets, posters, interactive activities and weekly lectures Workshops on soil‐transmitted helminth infections and prevention	Duration: 4 months	None stated	Usual school routine	School absence	Weak
Trinies et al. (2016), Mali	CBA	9−12 years old boys and girls	200 schools; 9730 participants; 53% boys	Water access in school Improved latrine facilities in school Provision of handwashing containers Hygiene kits in schools	Duration: 1 school year	None stated	No intervention	School absence	Weak
*Integrated multi‐component intervention*									
Barbosa Filho et al. (2019), Brazil	CRCT	11−18 years old boys and girls	6 schools; 1 085 participants; 52% boys	Three components: teacher training, health education and environmental changes Teacher training focused on healthy/unhealthy lifestyle, addressing physical activity, prevention of alcohol and tobacco consumption, and the relationship of these behaviours with the school environment and academic performance Supervised 10–15 min physical activity sessions, provision of sports equipment, banners, newsletters and pamphlets for students	Duration: 4 months	Socioecological theory, social cognitive theory and Health Promoting Schools	No intervention	Fruit juice intake, fruit intake, vegetable intake, soft drinks intake, savoury foods intake and sweet intake	Weak
Ferreira da Costa et al. (2014) and de Sousa et al. (2014), Brazil	CRCT	15−24 years old boys and girls	10 schools; 989 participants; 44% boys	Monthly 1 h long classroom sessions on eating habits and food consumption for students by nutritionist Sessions included playing games, staging theatre sketches, watching movies and puppet shows, and writing and drawing contests Weekly distribution of three types of seasonal fruit during school lunch Personnel engagement and training with school employees Healthy school day, posters, newsletter, website, bike racks, healthy snack day, weekend activities A set of messages for families in the form of illustrated booklets and recipes	Duration: 6 months	USDHHS/CDC's Physical Activity Evaluation Handbook	No intervention	Obesity, waist circumference, waist‐to‐height ratio, consumption of foods and beverages	Weak
Erismann et al. (2017), Burkina Faso	CRCT	8−14 years old boys and girls	8 schools; 360 participants; 51% boys	School garden: provision of seeds and small gardening tools and agricultural training to teachers and principals WASH intervention activities Educational behaviour change strategy for teachers, principals, and community members Treatments for children found anaemic or infected with intestinal parasites	Duration: 7 months	Vegetables Go to School	No intervention	Anthropometric indices (undernutrition, stunting, thinness, and overweight), Anaemia, BMI and height *z*‐score and haemoglobin	Moderate
Florence et al. (2020), Kenya	CRCT	15−18 years old boys and girls	4 schools; 222 participants; 49% boys	Physical education component: Weekly theory (40 min each) and practical sessions (60 min each) Classroom sessions focused on different types of physical activity, health benefits of physical activity, and ways of reducing sedentary time using lecture, role‐play, and group discussions Physical activity session: warm‐up and specific exercises 40 min weekly nutrition education sessions using class discussions, question and answer method, role play, and demonstrations	Duration: 15 weeks follow‐up: 8 weeks and 6 months after baseline	None stated	Regular 40 min of physical education (P.E) class sessions without a gym instructor	BMI *z*‐score, and waist circumference	Moderate
Gall et al. (2018) and Müller et al. (2019), South Africa	CRCT	Boys and girls from Grade 4	8 schools; 746 participants; 49% boys	Regular physical activity classes including two physical education lessons per week One weekly moving‐to‐music class Regular in‐class activity breaks incorporated into the main school curriculum School infrastructure adaptation to create a low‐cost ‘physical activity‐friendly’ environment Classroom‐based nutrition education	Duration: 20 weeks	None stated	Regular physical education	BMI *z*‐score, stunting, anaemia, concentration, attention and academic performance	Moderate
Jemmott et al. (2011, 2019), South Africa	CRCT	Boys and girls from Grade 6	18 schools; 1057 participants; 51% boys	12 one hour‐long modules, with 2 modules delivered during each of six sessions on 6 consecutive school days Six comic workbooks for students Use of interactive exercises, games, brainstorming, roleplaying and group discussions Aerobic, strength‐building and flexibility‐increasing exercises in school Homework assignments to do with parents	Duration: 13 months follow‐up: 3, 6, 12, 42 and 54 months post‐intervention	Social cognitive theory and theory of planned behaviour	HIV/STD risk‐reduction intervention	Fruit and vegetable consumption	Moderate
Leme et al. (2016, 2018), Brazil	CRCT	14−18 years old girls	10 schools; 253 participants	Ten activities: enhanced physical education classes, physical activities during recess, weekly nutritional and physical activity messages, nutrition and physical activity handbooks, interactive seminars, nutrition workshops, parents’ newsletters, text messages, and dietary and physical activity diaries for adolescent girls Obesity prevention programme with nutrition, physical and sedentary behaviours components	Duration: 6 months	Social cognitive theory	No intervention	BMI, BMI *z*‐score and waist circumference	Strong
Levy et al. (2012), Mexico	CRCT	10−13 years old boys and girls	60 schools; 1020 participants; 49% boys	Six nutrition and physical activity workshops using a puppet theatre for students A 2‐day workshop with teachers School‐level activities to change school diet and nutrition environment A recipe calendar for parents	Duration: One year	None stated	No intervention	Change in overweight and obesity status	Strong
Morales‐Ruán et al. (2014), Mexico	CRCT	Boys and girls from Grade 5	10 schools; 1020 participants; 49% boys	Nutrition and physical activity workshops using a puppet theatre for students 15 min of physical activity for 2 days per week Twice a week organized games during recess 2‐day awareness workshops for teachers and school cooperative staff Nutrition and physical activities such as the sale of fruits, vegetables, and pure water at school Broadcasting of audio spots three times per week during recess, and distribution of banners and recipe calendars	Duration: 6 months	None stated	No intervention	Prevalence of overweight and obesity	Moderate
Saraf et al. (2015), India	CRCT	Boys and girls from Grades 6 and 7	40 schools; 2074 participants; 53% boys	Classroom‐level: Health education lectures, flash films, flipchart sessions, and peer group discussions School‐level: Formation of the school health committee, formulation of a school action plan, adoption of policies, display of posters, and competitions Family/community‐level: Holiday assignments, school rally, and distribution of pamphlets	Duration: 9 months	Health promoting schools	No intervention	Knowledge of healthy and unhealthy foods and consumption practices and school food policy and environment	Moderate
Shirazi et al. (2019), Iran	CRCT	13−15 years old girls	2 schools; 230 participants	Eightone‐hour long weekly nutrition education sessions for adolescents Two weekly sessions for parents and teachers A supportive group of parents and teachers for encouraging the adolescents healthy nutritional behaviours Meetings with decision‐makers and stakeholders' Participatory homework for parents and adolescents 10 text and video messages to the parents	Duration: 8 weeks follow‐up: 6 months post‐intervention	Social cognitive theory	Routine monthly lectures covering the benefits of healthy diets by health teachers at schools	Dietary behaviours	Weak
Shrestha et al. (2020), Nepal	CRCT	8−17 years old boys and girls	12 schools; 682 participants; 53% boys	School garden with education once a week during a 90‐min long class with an emphasis on learning by doing in the school gardens Complementary interventions such as health promotion activities, posters, nutrition booklet, and hand‐outs for students; demonstration of handwashing with soap, audio‐visual material, construction of at least three latrines and 6–12 handwashing stations per school, and weekly education programmes for caregivers	Duration: 12 months	None stated	No intervention	Prevalence of stunting, thinness and anaemia	Moderate
Singhal et al. (2010), India	CRCT	15−17 years old boys and girls	2 schools; 201 participants; 60% boys	Health information through lectures and discussions Promotion of physical activity Weekly individual counselling sessions a nutritionist Policy level changes in the school such as a change in the school menu Involvement of teachers and parents Other activities to promote a healthy lifestyle such as planning tiffin, quiz, and extempore competitions Training of student volunteers to sustain the programme activities	Duration: 6 months	Dietary Guidelines for Indians	No intervention	Height, weight, BMI, waist circumference, waist to hip ratio and waist to height ratio	Weak
Styen et al. (2015), South Africa	CRCT	Boys and girls from Grades 4−6	16 schools; 998 participants; sex distribution not mentioned	Healthy school nutrition policies Nutrition education support Improving school shops Encouraging the promotion of healthy foods at special events Initiation of vegetable gardens at schools	Duration: 3 years	Socioecological model	A booklet with ‘tips’ for healthy schools and a guide to resources	Dietary diversity, fat intake and sugar intake	Weak
Thakur et al. (2016), India	CRCT	13−15 years old boys and girls	4 schools; 462 participants; 71% boys	Fortnightly health education sessions and audio‐visual displays Maintenance of lifestyle diaries Suitable dietary recommendations to the parents Ensuring one period of physical activity at school daily School canteen menu change Parental involvement to reduce television watching hours	Duration: 20 weeks	Health promoting schools	Information about diet and physical activity following baseline assessment	Height, weight, BMI, waist and hip circumference and waist‐hip ratio	Strong
Z. Wang et al. (2018), China	CRCT	Boys and girls from Grades 4 and 7	48 schools; 10,091 participants; 53% boys	Weekly 45‐min long classroom sessions on obesity and physical activity Blackboard writings, posters, slogans, and news leaflets for students and parents One health class per semester for parents Homework to complete between students and parents Other activities such as no TV week, walk to school week, contests, and live shows	Duration: 1 year	None stated	No intervention	BMI, BMI *z*‐score, overweight and obesity	Strong
F. Xu et al. (2015), China	CRCT	Boys and girls from Grade 4	8 schools; 1182 participants; 56% boys	Monthly 30‐min long classroom session on physical education and a healthy diet School environment support (posters, competitions, drama, etc.,) Health classes for parents/guardians Fun programmes/events for students	Duration: 2 semesters	Theory of triadic influence and the Comprehensive School Health Programme Model	Routine health education practice	BMI, and dietary habits	Strong
H. Xu (2017), China	CRCT	Boys and girls from Grades 1 to 5	38 schools; 8573 participants; 51% boys	Nutrition education intervention: Six 40‐min long courses for students; four for teachers and two for the parents Distribution of cartoon pamphlets and posters for students Twice a day 10‐min physical activity session	Duration: 2 semesters	None stated	No intervention	BMI, BMI *z*‐score, body fat %, waist circumference and overweight and obesity	Strong
Alaofè et al. (2009), Benin	CBA	12−17 years old girls	2 schools; 68 participants	Four weekly nutrition education lessons on sources and functions of iron, prevalence, and symptoms of iron deficiency, followed by quizzes and discussion Increase in the content and bioavailability of dietary iron in the cafeteria menu for 22 weeks	Duration: 26 weeks	None stated	No intervention	BMI, anaemia and iron‐deficiency anaemia	Weak
Tamiru et al (2016a, 2016b), Ethiopia	CBA	Boys and girls from Grades 1 to 8	4 schools; 1000 participants; 50% boys	Peer‐led health education School media and clubs Nutrition messages using artistic skills School‐community health committee Community events	Duration: 8 months	None stated	No intervention	Height *z*‐score, dietary diversity, and consumption of animal source food	Weak
Wei et al. (2019), Zambia	CBA	4−16 years old boys and girls	14 schools; 367 participants; 48% boys	Training for teachers to deliver health lessons to students, perform basic first aid, recognize common illnesses among students, identify warnings signs and refer these cases to skilled medical attention, and improve WASH conditions Bi‐annually health screening for students	Duration: 1 school year	None stated	No intervention	Weight, thinness, stunting, overweight, health knowledge and self‐reported absenteeism	Weak

Abbreviations: CBA, controlled before‐after study; CRCT, cluster randomized controlled trial.

### 3.2 Study design, setting and population

The 58 intervention studies included 41 CRCTs, 16 CBA studies and one individually randomized controlled study (Table 1).

The 58 intervention evaluations were set in 20 countries. Of these, 51.7% studies were conducted in Asia (nine each from China and Iran, five from India, three from Malaysia and one each from Laos, Nepal, Palestine and Turkey); 27.6% were from South America and the Caribbean countries (11 from Brazil, two from Mexico and one each from Colombia, Ecuador, Peru); and 20.7% were from Africa (three each from South Africa and Kenya, two from Ethiopia and one each from Burkina Faso, Benin, Mali, Zambia; Table 1).

Participants' ages ranged from 5 to 24 years (Grades 1−12). There were 31 interventions aimed at adolescents aged 10−19, 22 interventions targeted children and younger adolescents aged 5−14, three interventions were aimed at children and adolescents aged 5−19, and two targeted adolescents and young adults aged 15−24. Participants ranged from 60 to 10,091. Most CRCTs used the school as a randomization unit, ranging from 2 to 200 clusters.

### 3.3 Intervention characteristics

#### 3.3.1 Intervention duration

Twenty‐two interventions ran between 1 week and 5 months including five interventions of a month or lesser duration. Twenty‐two interventions ran between 6 and 11 months. Seven interventions ran for a year, and one intervention each for 2 and 3 years (Table 1). For five studies, it was not possible to determine the intervention duration (Hosseini et al., 2015; Karimi‐Shahanjarini et al., 2013; Keshani et al., 2019; Leventhal et al., 2016;  Sharif Ishak et al., 2020; Taghdisi et al., 2016).

#### 3.3.2 Theoretical framework for the interventions

Twenty‐six of the 58 intervention studies explicitly mentioned using the theoretical framework to inform their intervention. A total of 14 different theories were identified, with a few studies informed by more than one theoretical model. The most cited theory was the social cognitive theory (Barbosa Filho et al., 2019; Chagas et al., 2020; Jemmott et al., 2011, 2019; Leme et al., 2016, 2018; Najimi & Ghaffari, 2013; Shirazi et al., 2019), followed by the health promoting schools (HPS) framework (Alaofe et al., 2009; Barbosa Filho et al., 2019; Saraf et al., 2015; Thakur et al., 2016; D. Wang, Stewart, Chang & Shi, 2015; D. Wang, Stewart & Chang, 2015); and theory of planned behaviour (Jemmott et al., 2011, 2019; Karimi‐Shahanjarini et al., 2013; Taghdisi et al., 2016).

#### 3.3.3 Intervention focus

**Figure 2:**
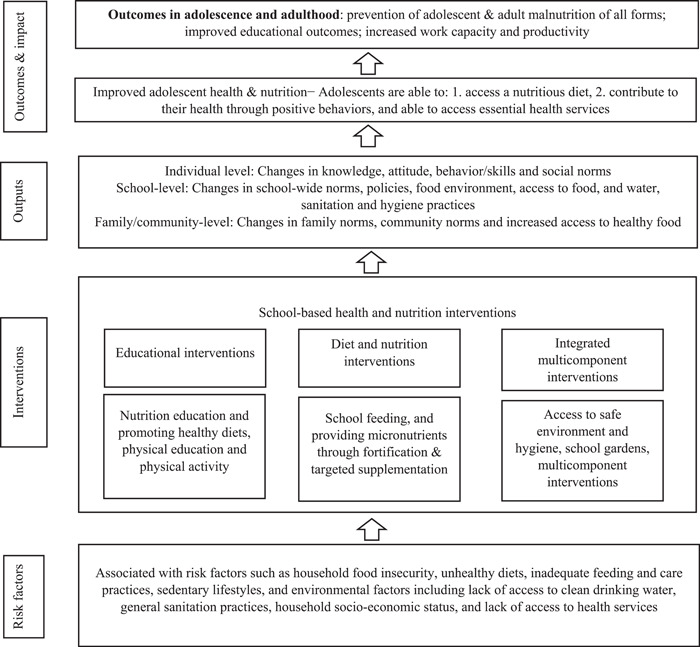
Framework for evidence synthesis of school‐based interventions of adolescent double burden of malnutrition

We developed a conceptual framework based on existing guidelines and recommendations (Shinde et al., 2021) that identified three types of school‐based health and nutrition interventions (Figure 2). These interventions included health education interventions (nutrition education, physical education, physical activity), diet and nutrition interventions and integrated multi‐component interventions (two or more of the following: nutrition education, physical education, physical activity, health checks, growth monitoring, deworming, school gardens, school food and nutrition environment and policies implementation and evaluation and WASH interventions). These interventions target knowledge, attitudes and behaviours about healthy diets at the individual level, norms, policies, and the food environment at the school level, and norms and access to healthier foods at the family and community levels. As a result, these changes will have an impact on behaviour related to diet, hygiene, cooking and food hygiene skills, as well as performance and participation in school. Ultimately, these behaviour changes would lead to desired health and educational outcomes, including a reduction in all types of malnutrition and improved academic performance.

Twenty‐seven publications evaluated 23 nutrition education interventions, 26 publications evaluated 21 integrated multi‐component interventions, 8 publications evaluated physical activity interventions, 5 examined WASH initiatives, and 1 studied the impact of food subsidy (Table 1).

#### 3.3.4 Intervention curriculum

The specific content and activities of interventions varied according to the health and nutrition topics targeted (Table 1). The following provides a summary of content and the types of activities undertaken, changes to the school environment, involvement of families and communities, and any other supplementary activities undertaken within the broad framework of identified interventions.

##### Nutrition education

Nutrition education interventions were primarily intended to increase knowledge and change the attitude towards diet and nutrition by providing information about topics such as balanced diet and nutrition, food pyramid, unhealthy and healthy food groups, benefits of eating fruits and vegetables, and prevention of anaemia and obesity (Table 1). These interventions addressed behavioural change by teaching skills such as setting goals, selecting nutritious food items, avoiding unhealthy foods, keeping a food diary and monitoring progress.

Nutrition education was delivered through weekly lectures (ranging between 30 and 90 min) and interactive activities such as group discussions, role‐plays, games, quizzes, puppet shows, movies, distribution of information materials and homework assignments. Complementary strategies included demonstrations of recipe preparations, food tasting sessions and food label reading training.

A few interventions engaged teachers and families through brief sessions lasting 45–60 min (Akdemir et al., 2017; Chagas et al., 2020; Keshani et al., 2019; Najimi & Ghaffari, 2013; Taghdisi et al., 2016; D. Wang, Stewart & Chang, 2015; D. Wang, Stewart, Chang & Shi, 2015). These sessions focused on providing information about the content of the intervention, advice on how to support intervention messages at home/school, and how to help adolescents adopt healthy eating skills and remain committed to reaching health goals. The interventions also used information and communication strategies such as the distribution of information materials (booklets, brochures, recipe guides), and frequent phone calls to parents providing feedback on their child's nutritional status.

Interventions were mostly conducted by the investigators themselves (Akdemir et al., 2017; Dansa et al., 2019; Shen et al., 2020; Sichieri et al., 2008, 2012), trained teachers (Chagas et al., 2020; Cunha et al., 2013; D. Wang et al., 2014; D. Wang, Stewart & Chang, 2015; D. Wang, Stewart, Chang & Shi, 2015; Yusoff et al., 2012, 2013), or nutrition experts (Amani & Soflaei, 2006; Taghdisi et al., 2016). Trained peer educators (Leventhal et al., 2016) and university undergraduate students (Ghray et al., 2013) delivered one intervention each.

##### Physical activity

The physical activity interventions consisted mainly of physical training classes led by physical education teachers or external instructors (Table 1). These classes ranged in frequency and duration (e.g., 30−90 min sessions twice a week to 5 days a week) of moderate to vigorous physical activities structured into the warm‐up, main part and cool‐down segments. Classes included aerobic exercises, dance sessions or sports. Few studies included complementary classroom‐based education sessions that addressed sedentary behaviour and the importance of physical activity (Andrade et al., 2014; Ochao‐Aviles et al., 2017; Guimarães et al., 2017; Li et al., 2014).

A few interventions also utilized promotional strategies including expert presentations, workshops, poster exhibitions, and brochure distributions (Andrade et al., 2014; Gutiérrez‐Martínez et al., 2018; Leong et al., 2015; Li et al., 2014; Ochoa‐Avilés et al., 2017). The involvement of family members, teachers and community members was limited. Physical activity sessions with parents and adolescent girls were included in two studies (Kargarfard et al., 2012; Li et al., 2014). Furthermore, workshops and phone text messages promoting healthy eating and exercise were provided to family members (Andrade et al., 2014; Gutiérrez‐Martínez et al., 2018; Ochoa‐Avilés et al., 2017).

##### WASH intervention

The WASH interventions focused primarily on two components: (1) infrastructure provision; and (2) behavioural changes (Table 1). The infrastructure component consisted of building toilets and handwashing stations and providing water, handwashing and sanitation facilities. The behaviour change component included the practice of group handwashing with soap at critical times, routine cleaning and maintenance of toilets, garbage collection and school compound maintenance. A few interventions also included health hygiene education using booklets, posters, hygiene kits and interactive activities on soil‐transmitted helminth transmission and prevention (Thériault et al., 2014; Trinies et al., 2016).

##### Nutrition subsidy

In one study, nutrition subsidies were used to reduce malnutrition among school children (Chen et al., 2019). School accounts received nutrition subsidies to use for nutrition‐related expenses in two different ways. The principals of one type of school were instructed to use the subsidy to reduce child malnutrition, and the principals of the other type of school were instructed to use the subsidy to reduce anaemia in students. For reversing the anaemic status, the second group would also receive monetary incentives.

##### Integrated multi‐component interventions

Several studies implemented a combination of two or more interventions to improve nutritional status and dietary habits in adolescents (Table 1). Hybrid strategies of nutrition education and physical activity (Barbosa Filho et al., 2019; Florence et al., 2020; Gall et al., 2019; Jemmott et al., 2011, 2019; Leme et al., 2016, 2018; Müller et al., 2019; Morales‐Ruan et al., 2014; Singhal et al., 2010; Thakur et al., 2016; Z. Wang et al., 2018; Xu et al., 2015, 2017) were used to improve knowledge of diet and nutrition, physical activity and other noncommunicable disease risk factors such as mental health, tobacco and alcohol consumption. School gardens were used to improve adolescents' knowledge of nutrition and preferences for vegetables (Erismann et al., 2017; Shrestha et al., 2020; Styen et al., 2015). A few integrated multi‐component interventions also included activities related to WASH (Erismann et al., 2017; Shrestha et al., 2020; Wei et al., 2019), and screening and treatment of students who were anaemic or infected with intestinal parasites (Trinies et al., 2016; Wei et al., 2019).

Additionally, several integrated multi‐component interventions included activities to improve school food and nutrition environment and policies (Alaofè et al., 2009; Barbosa Filho et al., 2019; Ferreira da costa et al., 2014; Ferreira da Sousa et al., 2014; Gall et al., 2018; Shamah Levy et al., 2012; Morales‐Ruan et al., 2014; Müller et al., 2019; Saraf et al., 2015; Shirazi et al., 2019; Shrestha et al., 2020; Singhal et al., 2010; Styen et al., 2015; Tamiru et al., 2016a, 2016b; Thakur et al., 2016; F. Xu et al., 2015). Activities included modifying school infrastructure to create a ‘healthy food and physical activity‐friendly’ environment, coordinating with the school health committee, developing and implementing action plans, providing information to students and parents and organizing school‐ and community‐level events.

Participation of students in intervention activities was encouraged through seminars, workshops, discussions and peer‐ or volunteer‐led health education and interactive activities (Ferreira da costa et al., 2014; Ferreira de Sousa et al., 2014; Jemmott et al., 2011, 2019; Levy et al., 2012; Morales‐Ruan et al., 2014; Tamiru et al., 2016a, 2016b; Z. Wang et al., 2018; F. Xu et al., 2015). Teachers were engaged in school health committees, design and implementation of school‐level policies, school gardening, and educational and physical activities (Erismann et al., 2017; Levy et al., 2012; Morales‐Ruan et al., 2014; Saraf et al., 2015; Shirazi et al., 2019; Shrestha et al., 2020; Singhal et al., 2010; Tamiru et al., 2016a, 2016b; Wei et al., 2019). Parents were engaged through workshops, educational materials, text and video messages and home assignments with their children (Jemmott et al., 2011, 2019; Leme et al., 2016, 2018; Levy et al., 2012; Saraf et al., 2015; Shirazi et al., 2019; Shrestha et al., 2020; Singhal et al., 2010; Xu et al., 2015, 2017).

### 3.4 Methodological quality of included studies

Table 1 presents the overall methodological quality of the included studies (details are in Supporting Information: Table S1). Of the 58 intervention evaluations, 10 were classified as strong in quality, 12 as moderate, and the remaining as weak. Twenty‐two moderate to strong quality studies included 13 integrated multi‐component intervention studies, 4 nutrition education intervention studies, 3 WASH intervention studies, and 1 study each on food subsidy and physical activity. Weak ratings were mainly due to high dropout rates, and missing data on components under consideration, methods of randomization, selection bias, blinding of assessors to intervention allocation and confounding.

### 3.5 Main findings of interventions

Table 2 provides a summary of the intervention effects based on the direction of the effects by intervention type. An Supporting Information: Table S2 provides study‐by‐study intervention effects. Overall, 16 out of 21 studies of integrated multi‐component interventions (76%) and 12 out of 23 studies of nutrition education (52%) reported an improvement in at least one nutrition or diet‐related outcome. Among the eight studies of physical activity intervention, three studies (38%) reported improvement in nutrition or diet‐related outcomes, while one study (13%) reported improvement in educational outcomes. In five studies of WASH intervention, only educational outcomes were reported. Sixteen of the 22 studies (73%) with moderate to strong methodological quality, 11 of which used multi‐component interventions, reported significant intervention effects on at least one nutrition or diet‐related outcome. Below, we provide a narrative overview of outcomes according to the types of interventions.

**Table 2:** Summary of results of school‐based health and nutrition interventions targeting multiple forms of nutrition of adolescents in low‐ and middle‐income countries

Outcome	School‐based health and nutrition interventions											
	Nutrition education (*n* = 23)			Physical activity (*n* = 8)			WASH intervention (*n* = 5)			Integrated multi‐component intervention (*n* = 21)		
	# of studies assessing outcome	# of studies with no effect	# of studies with evidence of effect	# of studies assessing outcome	# of studies with no effect	# of studies with evidence of effect	# of studies assessing outcome	# of studies with no effect	# of studies with evidence of effect	# of studies assessing outcome	# of studies with no effect	# of studies with evidence of effect
Nutritional status												
Weight	3	2	1	1	1	‐	‐	‐	‐	3	2	1
Height	1	1	‐	‐	‐	‐	‐	‐	‐	2	1	1
Body mass index (BMI)	5	5	‐	3	‐	3	‐	‐	‐	8	7	1
BMI *z*‐score	3	2	1	2	2	‐	‐	‐	‐	6	2	4
Height *z*‐score	1	1	‐		‐	‐	‐	‐	‐	2	2	‐
Waist circumference	2	2	‐	1	1	1	‐	‐	‐	6	3	3
Hip circumference	1	1	‐	‐	‐	‐	‐	‐	‐	1	1	‐
Waist height ratio	1	1	‐	‐	‐	‐	‐	‐	‐	2	1	1
Waist hip ratio	‐	‐	‐	‐	‐	‐	‐	‐	‐	2	1	1
Abdominal skinfold	‐	‐	‐	1	1	‐	‐	‐	‐	‐	‐	‐
Total fat percentage	1	1	‐	3	3	‐	‐	‐	‐	1	1	‐
Fat‐free mass percentage	‐	‐	‐	1	‐	1	‐	‐	‐	‐	‐	‐
Total undernutrition	1	‐	‐		‐	‐	‐	‐	‐	1	1	‐
Overweight and obesity	2	1	1		2/2	‐	‐	‐	‐	6	3	3
Obesity	‐	‐	‐		‐	‐	‐	‐	‐	2	1	1
Stunting	‐	‐	‐		‐	‐	‐	‐	‐	4	3	1
Thinness	‐	‐	‐		‐	‐	‐	‐	‐	3	2	1
Haemoglobin	3	1	2		‐	‐	‐	‐	‐	1	1	‐
Serum ferritin	1	1	‐		‐	‐	‐	‐	‐	‐	‐	‐
Anaemia	‐	‐	‐		‐	‐	‐	‐	‐	4	2	2
Diet‐related outcomes												
Nutritional knowledge	9	3	6	1	‐	1	‐	‐	‐	1	‐	1
Knowledge of healthy eating	1	1	‐	‐	‐	‐	‐	‐	‐	‐	‐	‐
Attitude towards nutrition	‐	‐	‐	1	‐	1	‐	‐	‐	‐	‐	‐
Self‐efficacy	2	1	1	‐	‐	‐	‐	‐	‐	‐	‐	‐
Diet quality	2	1	1	‐	‐	‐	‐	‐	‐	‐	‐	‐
Diet behaviour	4	2	2	‐	‐	‐	‐	‐	‐	‐	‐	‐
Dietary diversity	2	2	‐	‐	‐	‐	‐	‐	‐	2	1	1
Breakfast intake	2	2	‐	1	1	‐	‐	‐	‐	1	‐	1
Fruit intake	5	1	4	‐	‐	‐	‐	‐	‐	5	2	3
Vegetable intake	5	‐	5	‐	‐	‐	‐	‐	‐	6	2	4
Fruit and vegetable intake	2	1	1	1	1	‐	‐	‐	‐	‐	‐	‐
Fried food intake	1	‐	1	‐	‐	‐	‐	‐	‐	3	1	2
Soft‐drinks intake	4	1	3	‐	‐	‐	‐	‐	‐	3	2	1
Educational and cognitive outcomes												
School absence	‐	‐	‐	‐	‐	‐	5	5	‐	1	1	‐
School enrolment	‐	‐	‐	‐	‐	‐	1	1	‐	‐	‐	‐
School dropout	‐	‐	‐	‐	‐	‐	1	1	‐	‐	‐	‐
Grade progression	‐	‐	‐	‐	‐	‐	1	1	‐	‐	‐	‐
Academic performance	‐	‐	‐	‐	‐	‐	‐	‐	‐	1	‐	1
Concentration and attention	‐	‐	‐	‐	‐	‐	‐	‐	‐	1	1	‐
Digit span	‐	‐	‐	‐	‐	‐	‐	‐	‐	1	‐	1
Word and colour identification	‐	‐	‐	1	‐	1	‐	‐	‐	‐	‐	‐
Chinese language performance	‐	‐	‐	1	‐	1	‐	‐	‐	‐	‐	‐
English language performance	‐	‐	‐	1	‐	1	‐	‐	‐	‐	‐	‐
Mathematics performance	‐	‐	‐	1	1	‐	‐	‐	‐	‐	‐	‐

#### 3.5.1 Nutrition education

The majority of nutrition education intervention studies assessed nutrition and diet‐related outcomes however, there were no reports of educational outcomes. (Table 1). Five of the studies that measured BMI found no improvement (Akdemir et al., 2017; Chagas et al., 2020; Cunha et al., 2013; Shen et al., 2020; Sichieri et al., 2012), while one out of three studies found significant improvement in BMI *z*‐score (Dansa et al., 2019; Sharif Ishak et al., 2020; Shen et al., 2020). Of the two studies that measured obesity and overweight, one reported a significant reduction in obesity and overweight, while the other did not (Akdemir et al., 2017; Sichieri et al., 2012). A significant increase in haemoglobin levels was observed in two out of three studies (Amani & Soflaei, 2006; Chen et al., 2019; Yusoff et al., 2013).

In nine studies evaluating nutritional knowledge, six reported positive results (Amani & Soflaei, 2006; Ghray et al., 2013; Hosseini et al., 2015; Keshani et al., 2019; D. Wang et al., 2013; Yusoff et al., 2013), while three did not (Chagas et al., 2020; Fonseca et al., 2019; Leventhal et al., 2016). The average vegetable intake increased in five of the five studies that evaluated it (Brito Beck da Silva et al., 2015; Lin et al., 2017; Najimi & Ghaffari, 2013; Taghdisi et al., 2016; D. Wang, Stewart & Chang, 2015), the average fruit intake increased in four of the five studies (Brito Beck da Silva et al., 2015; Cunha et al., 2013; Lin et al., 2017; Najimi & Ghaffari, 2013; Taghdisi et al., 2016), and the soft drink consumption significantly reduced in three out of four studies (Brito Beck da Silva et al., 2015, 2019; Cunha et al., 2013; D. Wang, Stewart & Chang, 2015). Nutrition education interventions also assessed attitudes towards healthy eating, diet quality, dietary diversity and fried food consumption.

#### 3.5.2 Physical activity

There were significant increases in BMI in three physical activity intervention studies (Kargarfard et al., 2012; Li et al., 2014; Telles et al., 2013), but no significant increases in BMI *z*‐scores in two other studies (Gutiérrez‐Martínez et al., 2018; Ochoa‐Avilés et al., 2017). In three studies, the total fat content did not change significantly (Guimarães et al., 2017; Gutiérrez‐Martínez et al., 2018; Leong et al., 2015, pp. 51, 51, 54). In two studies, the change in overweight and obesity was not significant (Li et al., 2014; Ochoa‐Avilés et al., 2017). In one study, the breakfast intake, and fruit and vegetable intake were assessed, but the changes were not significant (Ochoa‐Avilés et al., 2017). One physical activity intervention study (Zhang et al., 2019) reported improved academic performance in Chinese and English languages but a negative effect on mathematics skills.

#### 3.5.3 WASH interventions

There were no improvements in school absences in four studies of WASH intervention (Caruso et al., 2014; Chard et al., 2019; Freeman et al., 2012; Thériault et al., 2014), while one study reported significantly higher absenteeism rates in intervention schools than comparison schools (Trinies et al., 2016). One WASH intervention study also assessed the impact on school enrolment, school dropout and grade progression, but the findings were not significant (Chard et al., 2019). None of the WASH intervention studies assessed nutrition or diet‐related outcomes.

#### 3.5.4 Nutrition subsidy

One study of nutrition subsidy evaluated the impact on anthropometry and biomarkers (Chen et al., 2019). The nutrition subsidy with the anaemia reduction target, compared with the nutrition subsidy with general targets, increased students' haemoglobin concentrations by 4.49 g/L (*p* = 0.009), which translated into a 12‐percentage point reduction in anaemia prevalence (*p* = 0.03). The subsidy with anaemia reduction target also raised treated students' BMI *z*‐scores by 0.12 standard deviations (*p* < 0.01), decreasing their risk of being underweight by 4.1 percentage points (*p* < 0.1) compared with the general target condition.

#### 3.5.5 Integrated multi‐component interventions

Anthropometric outcomes were assessed most frequently by the integrated multi‐component interventions, but the results were generally inconclusive. There were no significant changes in BMI in seven out of eight studies (Alaofè et al., 2009; Ferreira de Sousa et al., 2014; Leme et al., 2018; Singhal et al., 2010; Thakur et al., 2016; Z. Wang et al., 2018; Xu et al., 2015, 2017), but four out of six studies addressing overweight and obesity reported a significant change in BMI *z*‐scores (Erismann et al., 2017; Florence et al., 2020; Leme et al., 2018; Müller et al., 2019; Z. Wang et al., 2018; H. Xu et al., 2017). Three of the six studies reported a significant reduction in waist circumference (Ferreira de Sousa et al., 2014; Florence et al., 2020; Leme et al., 2018; Singhal et al., 2010; Thakur et al., 2016; H. Xu et al., 2017). Three out of five studies reported significant reductions in overweight and obesity (Carmen morales‐Ruán et al., 2014; Erismann et al., 2017; Z. Wang et al., 2018; Wei et al., 2019; H. Xu et al., 2017); two out of four studies reported a significant reduction in anaemia (Alaofè et al., 2009; Erismann et al., 2017; Müller et al., 2019; Shrestha et al., 2020); and one study each reported significant reductions in stunting (Wei et al., 2019) and thinness (Shrestha et al., 2020).

Several studies of integrated multi‐component intervention evaluated diet‐related outcomes, but the results were mixed. A significant increase in vegetable intake was seen in four out of six studies (Barbosa Filho et al., 2019; Ferreira de Sousa et al., 2014; Jemmott et al., 2019; Shrestha et al., 2020; H. Xu et al., 2017), and a significant increase in fruit intake was seen in three out of five studies (Ferreira de Sousa et al., 2014; Jemmott et al., 2019; Saraf et al., 2015; Shirazi et al., 2019; Z. Wang et al., 2018). A significant reduction in fried food intake was observed in two out of three studies (Jemmott et al., 2019; Saraf et al., 2015; H. Xu et al., 2017) and a significant reduction in soft drinks intake in one out of three studies (Barbosa Filho et al., 2019; Ferreira de Sousa et al., 2014; H. Xu et al., 2017).

Two studies of multi‐component intervention also evaluated the impact on educational outcomes, with one study reporting no impact on student absenteeism (Ref) while the other study reporting a positive change in academic performance but no effect on concentration and attention (Müller et al., 2019).

## 4 DISCUSSION

### 4.1 Synthesis of findings

This systematic review aimed to synthesize the available evidence on the effects of school‐based interventions addressing multiple forms of malnutrition on nutrition and educational outcomes among adolescents in LMICs. The review included 68 articles involving 58 interventions. The methodological quality was limited in most studies, with about a third of studies having moderate to strong design. The majority of the interventions (64%) were focused on a single domain and just over one‐third of the interventions (36%) were integrated multi‐component interventions. The programme development of most of these interventions was not theory‐based. The results of the effectiveness on anthropometric outcomes of these interventions were inconsistent. Around half of the nutrition education studies and two‐thirds of integrated multi‐component intervention studies reported beneficial effects on at least one nutrition or diet‐related outcome. For example, 4 of 16 studies that evaluated BMI, 5 of 11 studies that reported BMI *z*‐scores, 6 of the 10 studies that evaluated overweight and obesity, 4 of 10 studies that reported waist circumference, 8 of the 11 studies that evaluated nutritional knowledge, 9 of the 11 studies that measured vegetable intake and 7 of the 10 studies that measured fruit intake found beneficial effects. WASH interventions have largely focused on evaluating educational outcomes and have not had any effect on the outcomes. Due to the heterogeneity of interventions and outcomes across studies, it is not possible to make definite statements about the overall effectiveness and quality of the evidence.

Just as the importance of integrated interventions to improve maternal and child nutrition is increasingly recognized (Keats et al., 2021), we expect that integrated interventions combining educational, curricular, and environmental elements will be more effective in addressing multiple forms of malnutrition among adolescents than single domain interventions. This is consistent with the previous evidence on WHO's HPS framework and the Nutrition‐Friendly School Initiative (NFSI) (Al‐Khudairy et al., 2017; Bleich et al., 2018; Brown et al., 2016; Langford et al., 2015; Saraf et al., 2012; WHO, 2001, 2017a, 2017b, 2021). Each of these approaches considers the socio‐ecological realities faced by adolescents and advocates the availability of health and nutrition services, a health curriculum, school‐wide policies, and an engaging school community. As some studies in the present review indicated, teachers had a role to play in providing interventions and an enabling environment in schools. Several school and home‐based activities were organized to engage parents, such as workshops, seminars, and information communication through written and digital materials. Despite considerable interest in teacher and parental involvement in these interventions, the effectiveness of these programmes is unclear and requires further investigation. This is primarily because these studies did not report the level of participation of teachers and parents or did not examine which components of the intervention package or the characteristics of the context influenced the outcome(s).

The importance of theoretical frameworks in addressing adolescent malnutrition has been emphasized in multiple global guidelines and recommendations (Bundy et al., 2017; Patton et al., 2016; WHO, 2017a). To make research findings more meaningful, acceptable and generalizable, these frameworks can explain how the intervention is conducted and ground it firmly in theoretical constructs. About one‐third of interventions in the review were based on theory. The nutrition and educational outcomes reported in these studies were equally inconsistent as those reported in studies without a theoretical framework. Additionally, evidence suggests that studies with longer duration interventions (more than 6 months) showed significant effects compared to studies with shorter duration interventions (Jacob et al., 2021). Duration of intervention is important because it allows embedding of the intervention components and their effect, as well as some sustainability of behavioural changes, thereby strengthening the evidence of the findings. The results of the current study, however, showed mixed findings. Of the 11 studies with one or more yearlong interventions, six had significant results on at least one outcome. Further, both single and multi‐component interventions produced positive effects on nutrition status mainly in studies with small sample sizes, which may not have sufficient power to detect effects. Many of these studies included both children and adolescents and did not focus exclusively on adolescents.

Health and education are linked bidirectionally (Patton et al., 2016). Higher education is associated with better access to health care and improved health and nutrition outcomes and practices among adolescents. Conversely, adolescents who are ill or undernourished are more likely to miss days of school, fall behind academically, and dropout of school (Bundy et al., 2017; Patton et al., 2016; WHO 2017b, 2021). In the present review, WASH intervention studies largely focused on school absenteeism as a measure of educational outcome. Our findings indicate that school‐based WASH interventions do not result in reduced absenteeism. Contrary to our findings, a systematic review of 38 studies of WASH interventions in low‐income countries found mixed impacts on student absenteeism (McMichael, 2019). Findings of a meta‐analysis of 19 school feeding studies based on a systematic review and meta‐analysis of 57 articles indicate a significant increase in the percentage of school days spent in school (2.6%; 95% CI = 1.2%, 3.9%; *p* < 0.001), but no significant impact on mathematical/arithmetic skills (mean difference in scores = 0.320; 95% CI = −0.148, 0.789; *p* = 0.018) (D. Wang et al., 2021). Furthermore, the effects of school‐based nutrition interventions on other educational outcomes such as academic performance, school drop‐out, and age‐appropriate verbal and numerical skills, are unclear in LMICS. In our view, the evaluation of school‐based health and nutrition interventions should also focus on evaluating their impact on educational outcomes.

Studies included in this review have some limitations worth discussing. While most of the studies utilized CRCT designs with large student populations, randomized schools in each arm were typically few, which could overestimate the intervention effects. Except for one study, allocation concealment and masking of participants and assessors were impossible because interventions were too obvious. Moreover, in multi‐component interventions, schools could modify interventions, resulting in heterogeneity of intervention activities and delivery and varying intervention doses. Further, interventions are rarely as effective in real‐world settings as they are in RCTs, where experts or investigators often carry out interventions (McCrabb et al., 2019). Almost all studies evaluated effectiveness shortly after the end of the intervention. In contrast, interventions might potentiate a process of long‐term habit change and long‐term effects, irrespective of nonsignificant short‐term effects (Herbert et al., 2018).

### 4.2 Strengths and limitations

In interpreting the results of this systematic review, it is important to consider its strengths and limitations. One of the strengths of this review is that it describes school‐based approaches to combat the DBM in adolescents from LMICs. Considering the increasing concern about multiple forms of malnutrition among children and adolescents in LMICs, we sought to understand how the integrated framework for interventions is being used. The use of the PRISMA and SWiM guidelines along with the conceptual framework to guide analysis and reporting ensures robustness and reproducibility. There are, however, some limitations to our work. First, our search strategy may have missed relevant multi‐disciplinary interventions addressing educational outcomes through specific databases such as Educational Resources Information Center database and Education Research Complete. Second, important information such as random allocation method, sociodemographic details of adolescents and characteristics of schools, selection bias, controlling of confounding, blinding of assessors, psychometric properties of data collection tools, coverage and quality of intervention activities, and adverse effects of the interventions were either omitted or poorly described in the original publications. Finally, meta‐analysis was not possible due to the heterogeneity of the interventions and outcomes.

### 4.3 Implications of findings

Despite limited evidence about school‐based health and nutrition programmes for adolescents; our review suggests that multi‐component integrated interventions are beneficial for nutrition and diet‐related outcomes in LMICs. Multiple forms of malnutrition are highly prevalent among adolescents in LMICs, and research on prevention attempts in education settings is limited. This study illustrates the need for further intervention efforts in developing countries. The possibilities of adapting global school health guidelines, such as HPS and NFSI, must be explored through rigorous, multi‐site, well‐designed studies of LIMCs. These interventions should incorporate school health policies, curriculum elements, health services, and the school environment, as well as student, parent and community engagement. In addition, schools and public spaces should provide adequate facilities for children and adolescents to engage in physical activity.

## 5 CONCLUSION

The current COVID‐19 outbreak is expected to exacerbate all forms of malnutrition worldwide, a setback against Sustainable Development Goal 2 of ending all forms of malnutrition by 2030. The governments, civil society organizations and donor agencies should develop innovative strategies, including digital interventions, to reach children and adolescents and provide the funds and resources necessary to implement and evaluate these strategies. The increasing prevalence of adolescent DBM in LMICs offers a unique and important opportunity for school‐based integrated action on malnutrition in all its forms. The focus of future studies should be on objective measures of body composition and dietary behaviour in addition to educational outcomes. Moreover, future studies should evaluate the benefits and costs of school‐based health and nutrition interventions. The findings from these interventions will contribute valuable data to policymaking in LMICs.

## AUTHOR CONTRIBUTIONS

Sachin Shinde, Dongqing Wang and Wafaie W. Fawzi developed the research questions and methodology. Sachin Shinde and Gretchen E. Moulton screened titles and abstracts. Sachin Shinde, Gretchen E. Moulton and Dongqing Wang screened full‐texts, extracted data and assessed risk of bias. Sachin Shinde performed statistical analysis. Sachin Shinde wrote the paper. All authors read and critically edited the paper. All authors approved the final paper.

## CONFLICT OF INTEREST

The authors declare no conflict of interest.

## Supporting information

Supporting information.

## Data Availability

The data sets generated during and/or analyzed during the current study are available from the corresponding author on reasonable request.
