# Supplementary material for: School‐based health and nutrition interventions addressing double burden of malnutrition and educational outcomes of adolescents in low‐ and middle‐income countries: A systematic review
Source: Matern Child Nutr. 2023 Mar 30;21(Suppl 1):e13437. doi: 10.1111/mcn.13437 (PMC12208896; doi:10.1111/mcn.13437)
Supplement: Supplementary file 1 — Supporting information. [file MCN-21-e13437-s001.docx]

**Online Supplementary Table 1**: Quality of included studies.

| **First author and year** | **Selection bias^a^** | **Study design^b^** | **Confounders^c^** | **Blinding^d^** | **Data collection methods^e^** | **Withdrawals and drop-out^f^** | **Overall quality** |
| --- | --- | --- | --- | --- | --- | --- | --- |
| **Nutrition education** | | | | | | | |
| Akdemir, 2017 [22] | Moderate | Moderate | Strong | Moderate | Strong | Moderate | Strong |
| Chagas, 2020 [23] | Strong | Strong | Weak | Weak | Weak | Weak | Weak |
| Cunha, 2013 [24] | Weak | Weak | Moderate | Weak | Moderate | Moderate | Weak |
| da Silva, 2019 [25] | Moderate | Moderate | Moderate | Moderate | Moderate | Weak | Moderate |
| Fonseca, 2019 [26] | Moderate | Weak | Moderate | Weak | Moderate | Weak | Weak |
| Karimi-Shahanjarini, 2013 [27] | Moderate | Moderate | Weak | Moderate | Moderate | Weak | Weak |
| Keshani, 2019 [28] | Moderate | Weak | Weak | Weak | Moderate | Weak | Weak |
| Leventhal, 2016 [29] | Moderate | Moderate | Weak | Weak | Weak | Weak | Weak |
| Lin, 2017 [30] | Moderate | Moderate | Moderate | Weak | Moderate | Strong | Moderate |
| Najimi, 2013 [31] | Weak | Weak | Weak | Weak | Moderate | Weak | Weak |
| Sichieri, 2009 and 2013 [32, 33] | Moderate | Strong | Moderate | Weak | Moderate | Moderate | Moderate |
| Toral, 2012 [34] | Moderate | Weak | Weak | Weak | Weak | Weak | Weak |
| Wang, 2013 & 2014 [35, 36] | Weak | Weak | Weak | Weak | Moderate | Moderate | Weak |
| Wang 2015a, & 2015b [37, 38] | Weak | Weak | Weak | Weak | Moderate | Moderate | Weak |
| Yusoff, 2012 & 2013 [39, 40] | Weak | Weak | Weak | Weak | Moderate | Moderate | Weak |
| Amani, 2006 [41] | Moderate | Weak | Weak | Weak | Moderate | Strong | Weak |
| Dansa, 2019 [42] | Weak | Weak | Weak | Weak | Moderate | Weak | Weak |
| da Silva, 2015 [43] | Weak | Weak | Moderate | Weak | Moderate | Weak | Weak |
| Ghrayeb, 2013 [44] | Weak | Moderate | Weak | Moderate | Moderate | Strong | Weak |
| Hosseini, 2015 [45] | Weak | Weak | Weak | Weak | Weak | Weak | Weak |
| Ishak, 2020 [46] | Weak | Weak | Moderate | Moderate | Weak | Weak | Weak |
| Shen, 2020 [47] | Weak | Weak | Moderate | Weak | Moderate | Moderate | Weak |
| Taghdisi, 2016 [48] | Weak | Weak | Weak | Moderate | Moderate | Moderate | Weak |
| **Physical activity** | | | | | | | |
| Andrade, 2014 and Ochoa-Avilés, 2017 [49, 50] | Moderate | Strong | Strong | Moderate | Moderate | Moderate | Strong |
| Gutiérrez-Martínez, 2018 [51] | Weak | Weak | Moderate | Weak | Moderate | Weak | Weak |
| Leong, 2015 [52] | Weak | Weak | Weak | Weak | Moderate | Weak | Weak |
| Telles, 2013 [53] | Weak | Weak | Weak | Weak | Moderate | Moderate | Weak |
| Guimarães 2017 [54] | Weak | Weak | Weak | Weak | Moderate | Weak | Weak |
| Kargarfard, 2012 [55] | Weak | Weak | Moderate | Weak | Moderate | Moderate | Weak |
| Li, 2014 [56] | Weak | Weak | Moderate | Weak | Moderate | Moderate | Weak |
| Zhang, 2019 [57] | Weak | Weak | Moderate | Weak | Moderate | Moderate | Weak |
| **Nutrition subsidy** | | | | | | | |
| Chen, 2019 [58] | Strong | Weak | Moderate | Moderate | Moderate | Moderate | Moderate |
| **WASH intervention** | | | | | | | |
| Caruso, 2014 [59] | Strong | Strong | Moderate | Moderate | Moderate | Strong | Strong |
| Chard, 2019 [60] | Strong | Strong | Moderate | Weak | Moderate | Moderate | Moderate |
| Freeman, 2012 [61] | Moderate | Moderate | Moderate | Moderate | Moderate | Moderate | Strong |
| Theriault, 2014 [62] | Moderate | Weak | Moderate | Moderate | Moderate | Moderate | Weak |
| Trinies, 2014 [63] | Moderate | Weak | Moderate | Moderate | Moderate | Moderate | Weak |
| **Integrated multicomponent intervention** | | | | | | | |
| Barbosa Filho, 2019 [64] | Weak | Strong | Weak | Weak | Moderate | Strong | Weak |
| da Costa, 2014 and de Sousa, 2014 [65, 66] | Weak | Moderate | Moderate | Weak | Weak | Weak | Weak |
| Erismann, 2017 [67] | Strong | Strong | Moderate | Moderate | Weak | Weak | Moderate |
| Florence, 2020 [68] | Strong | Strong | Moderate | Moderate | Weak | Weak | Moderate |
| Gall, 2018 and Müller, 2019 [69, 70] | Strong | Moderate | Moderate | Weak | Strong | Strong | Moderate |
| Jemmott III 2011 & 2019 [71, 72] | Moderate | Moderate | Moderate | Weak | Moderate | Moderate | Moderate |
| Leme 2016 and 2018 [73, 74] | Moderate | Moderate | Moderate | Moderate | Moderate | Moderate | Strong |
| Levy, 2012 [75] | Moderate | Moderate | Moderate | Moderate | Moderate | Strong | Strong |
| Morales-Ruán, 2014 [76] | Moderate | Moderate | Moderate | Weak | Moderate | Moderate | Moderate |
| Saraf, 2015 [77] | Moderate | Strong | Moderate | Moderate | Moderate | Weak | Moderate |
| Shirazi, 2019 [78] | Weak | Weak | Moderate | Weak | Moderate | Moderate | Weak |
| Shrestha, 2020 [79] | Moderate | Strong | Moderate | Moderate | Moderate | Moderate | Moderate |
| Singhal, 2010 [80] | Weak | Weak | Moderate | Weak | Moderate | Moderate | Weak |
| Styen, 2015 [81] | Moderate | Moderate | Moderate | Weak | Weak | Weak | Weak |
| Thakur, 2016 [82] | Moderate | Moderate | Moderate | Moderate | Moderate | Moderate | Strong |
| Wang, 2018 [83] | Moderate | Strong | Moderate | Moderate | Moderate | Moderate | Strong |
| Xu, 2015 [84] | Moderate | Strong | Moderate | Moderate | Moderate | Moderate | Strong |
| Xu, 2017 [85] | Moderate | Strong | Moderate | Moderate | Moderate | Moderate | Strong |
| Alaofè, 2009 [86] | Weak | Weak | Moderate | Weak | Weak | Moderate | Weak |
| Tamiru, 2016a & 2016b [87, 88] | Weak | Weak | Moderate | Moderate | Weak | Weak | Weak |
| Wei, 2019 [89] | Weak | Weak | Moderate | Weak | Moderate | Strong | Weak |

^a:^ Likelihood of bias due to the allocation process in an experimental study; participants are more likely to be representative of the target population

^b^: Study design and method of randomization clearly described

^c^: Confounders were controlled in the design (by stratification or matching) or the analysis. If the allocation to intervention and control groups is randomized, the authors must report that the groups were balanced at baseline with respect to confounders

^d^: Assessors are blinded to intervention allocation

^e^: Tools for outcome measures are clearly described

^f^: Numbers and reasons for withdrawals and drop-outs are described

**Online Supplementary Table 2**: Effects of school-based interventions targeting multiple forms of nutrition of adolescents in low- and middle-income countries.

| **First author and year** | **Intervention group sample size** | **Control group sample size** | **Change in intervention** | **Change in control** | **Difference between change in intervention and control** | **Intervention effects as reported in primary studies** |
| --- | --- | --- | --- | --- | --- | --- |
| **Nutrition education** | | | | | | |
| **Akdemir, 2017 [22]** |  |  |  |  |  |  |
| Protection from overweight/obese status | 475 | 453 |  |  |  | 1.04 (1.01, 1.06)** |
| Overweight/obesity to normal weight | 172 | 188 |  |  |  | 1.88 (1.09, 3.24)** |
| Body mass index (BMI; kg/m^2^) | 674 | 675 | 0.17 | 0.18 | 0.01 |  |
| Diet quality index | 674 | 675 | 1.35 | -0.17 | 1.52*** |  |
| **Chagas, 2020 [23]** |  |  |  |  |  |  |
| Knowledge of healthy eating | 117 | 202 | 0.5 | 0.3 |  |  |
| Nutritional knowledge | 117 | 202 | 0 | -1 |  |  |
| Perception of a healthy diet | 117 | 202 | 0.4 | 0.3 |  |  |
| Self-efficacy | 117 | 202 | -0.2 | 0.1 |  |  |
| **Cunha, 2013 [24]** |  |  |  |  |  |  |
| BMI (kg/m^2^) | 282 | 277 |  |  |  | -0.006 |
| Cookies/day | 282 | 277 |  |  |  | 0.13*** |
| Sodas/day | 282 | 277 |  |  |  | 0.13* |
| Juices/day | 282 | 277 |  |  |  | 0.03 |
| Beans/day | 282 | 277 |  |  |  | -0.01 |
| Fruits/day | 282 | 277 |  |  |  | -0.16* |
| **da Silva, 2019 [25]** |  |  |  |  |  |  |
| Weight (kg) | 285 | 314 | 2.86 | 2.68 |  | 0.97 (0.75) |
| BMI (kg/m^2^) | 285 | 314 | 0.39 | 0.39 |  | 0.26 (0.25) |
| Waist circumference (cm) | 285 | 314 | 1.11 | 2.00 |  | 1.00 (0.58) |
| Hip circumference (cm) | 285 | 314 | 0.29 | 1.23 |  | 0.35 (0.62) |
| Waist-to-height ratio | 285 | 314 | 0.00 | 0.01 |  | 0.00 (0.03) |
| Beans consumption | 428 | 467 |  |  |  | 1.43 (1.10, 1.86) |
| Soft drinks consumption | 428 | 467 |  |  |  | 0.65 (0.50, 0.84) |
| **Fonseca, 2019 [26]** |  |  |  |  |  |  |
| Dietary knowledge | 273 | 188 | 1.41 | 0.58 |  | -1.18 (-1.85, -0.51)** |
| Self-perceived diet quality | 273 | 188 | 0.13 | 0.46 |  | -0.36 (-0.92, 0.19) |
| Breakfast eating practice | 142 | 143 | 0.6 | 2.2 |  | 2.67 (0.68, 7.57) |
| **Karimi-Shahanjarini, 2013 [27]** |  |  |  |  |  |  |
| Unhealthy snack intake | IG1: 181  IG2: 189 | 220 | IG1: -0.59**  IG2:-1.17*** | 0.41 |  | IG1 vs C:  -1.20 (-3.29, 0.89)  IG2 vs C:  -2.09 (-4.14, -0.04)*** |
| Healthy snack intake | IG1: 181  IG2: 189 | 220 | IG1: -0.78  IG2:-0.77 | -0.67 |  | IG1 vs C:  -1.02 (-2.82, 0.78)  IG2+ vs C:  0.01 (-0.85, 0.87) |
| **Keshani, 2019 [28]** |  |  |  |  |  |  |
| Diet quality index | 164 | 148 | 1.39*** | -0.40 |  |  |
| Knowledge of nutrition | 164 | 148 | 7.76*** | 0.10 |  |  |
| **Leventhal, 2016 [29]** |  |  |  |  |  |  |
| Health knowledge | IG1: 847  IG2: 825  IG3:828 | 695 |  |  |  | IG1 vs C: 5.11***  IG2 vs C: 4.20***  IG3 vs C: 0.17 |
| Nutrition knowledge | IG1: 728  IG2: 745  IG3:744 | 659 |  |  |  | IG1 vs C: 0.13  IG2 vs C:-0.11  IG3 vs C: -0.03 |
| **Lin, 2017 [30]** |  |  |  |  |  |  |
| Fruit intake | IG1: 493  IG2: 449 | 471 | IG1: 1.43  IG2: 0.25 | -0.54 |  | IG1 v C: 1.43***  IG2 v C: 0.57* |
| Vegetable intake | IG1: 493  IG2: 449 | 471 | IG1: 1.05  IG2: 0.34 | -0.26 |  | IG1 v C: 0.92***  IG2 v C: 0.45** |
| **Najimi, 2013 [31]** |  |  |  |  |  |  |
| Fruit intake | 63 | 67 | 0.37*** | -0.11 | 0.48*** |  |
| Vegetable intake | 63 | 67 | 0.75*** | 0.06 | 0.69*** |  |
| **Sichieri, 2009 and 2013 [32, 33]** |  |  |  |  |  |  |
| Weight (kg) | 434 | 493 | 2.8 | 2.8 | -0.00009 |  |
| BMI (kg/m^2^) | 434 | 493 | 0.32 | 0.22 | 0.10 |  |
| Prevalence of overweight | 434 | 493 | 3.0 | 2.1 | 0.9 |  |
| Prevalence of obesity | 434 | 493 | 0.34 | 0.40 | 0.06 |  |
| Fruit juice | 434 | 493 |  |  |  | 0.16 (0.02, 0.30)* |
| **Toral, 2012 [34]** |  |  |  |  |  |  |
| Fruit and vegetable intake | 448 | 323 | -1.1 | -3.1 |  |  |
| **Wang, 2013, and 2014 [35, 36]** |  |  |  |  |  |  |
| Nutrition knowledge score | IG1: 62  IG2: 65 | 61 | IG1: 3.31***  IG2: 3.11*** | 1.3 |  |  |
| Eating behavior score | IG1: 62  IG2: 62 | 61 | IG1: 0.97***  IG2: 0.76*** | 0.38 |  |  |
| **Wang, 2015a and 2015b [37, 38]** |  |  |  |  |  |  |
| Drinking no soft drinks | 65 | 61 |  |  |  | 1.99 (1.31, 2.03)*** |
| Eating no desserts | 65 | 61 |  |  |  | 3.9 (2.4, 6.4)*** |
| Eating no fried foods | 65 | 61 |  |  |  | 3.63 (2.25, 5.85)*** |
| Fruit intake | 65 | 61 |  |  |  | 1.33 (0.87, 2.04) |
| Vegetable intake | 65 | 61 |  |  |  | 2.51 (1.40, 4.48)** |
| Drinking milk | 65 | 61 |  |  |  | 1.42 (0.87, 2.30) |
| Eating breakfast |  |  |  |  |  | 1.41 (0.91, 2.19) |
| **Yusoff, 2012 and 2013 [39, 40]** |  |  |  |  |  |  |
| Hemoglobin | 67 | 56 | 2.23 | -1.58 | 3.81*** |  |
| Nutrition knowledge | 156 | 126 | 18.03 | -0.5 | 18.53** |  |
| **Amani, 2006 [41]** |  |  |  |  |  |  |
| Nutritional knowledge score | 30 | 30 | 7.1*** | 0.7 |  |  |
| Lifestyle score | 30 | 30 | 0.1 | -0.3 |  |  |
| Food frequency score | 30 | 30 | 2.8* | -2.2 |  |  |
| Hemoglobin (g/dL) | 30 | 30 | -0.3 | -0.1 |  |  |
| Serum ferritin (ng/ml) | 30 | 30 | -3.1 | -3.0* |  |  |
| **Dansa, 2019 [42]** |  |  |  |  |  |  |
| Weight (kg) | 66 | 66 | 2.1*** | 0.1 |  |  |
| Height (cm) | 66 | 66 | 1.1 | 0.2 |  |  |
| BMI z score | 66 | 66 | 2.0*** | -1.0 |  |  |
| Height z score | 66 | 66 | -2.0 | 1.0 |  |  |
| Dietary diversity score | 66 | 66 | 0.98*** | -0.24 |  |  |
| **da Silva, 2015 [43]** |  |  |  |  |  |  |
| BMI (kg/m^2^) |  |  |  |  |  | Not reported |
| Beans consumption | 265 | 365 |  |  |  | 1.09 (0.87, 1.36) |
| Rice consumption | 265 | 365 |  |  |  | 1.11 (0.89, 1.39) |
| Legumes consumption | 265 | 365 |  |  |  | 1.18 (1.03, 1.37)* |
| Vegetable consumption | 265 | 365 |  |  |  | 1.17 (1.01, 1.35)* |
| Fruits consumption | 265 | 365 |  |  |  | 1.13 (0.93, 1.37) |
| Milk consumption | 265 | 365 |  |  |  | 1.14 (0.91, 1.44) |
| Candies consumption | 265 | 365 |  |  |  | 1.15 (0.92, 1.44) |
| Snacks consumption | 265 | 365 |  |  |  | 0.93 (0.73, 1.23) |
| Processes meats consumption | 265 | 365 |  |  |  | 1.16 (0.88, 1.52) |
| Soft drinks consumption | 265 | 365 |  |  |  | 1.10 (0.88, 1.37) |
| **Ghrayeb, 2013 [44]** |  |  |  |  |  |  |
| Knowledge of nutrition | 116 | 120 | 1.91*** | -0.10 |  |  |
| **Hosseini, 2015 [45]** |  |  |  |  |  |  |
| Knowledge of benefits of breakfast | 44 | 44 |  |  | 1.37** |  |
| Attitude towards breakfast consumption | 44 | 44 |  |  | 3.75** |  |
| Subjective norms towards breakfast consumption | 44 | 44 |  |  | 6.96*** |  |
| **Ishak, 2020 [46]** |  |  |  |  |  |  |
| BMI z score | 34 | 42 |  |  |  | -0.14 (-0.53, 0.24) |
| Waist circumference | 34 | 42 |  |  |  | -1.3 (-4.9, 2.3) |
| % of body fat | 34 | 42 |  |  |  | -0.7 (-2.0, 0.7) |
| Knowledge of a healthy lifestyle | 34 | 42 | 2.99 | 3.08 |  | 2.82 (0.86, 4.78)* |
| Attitude toward a healthy lifestyle | 34 | 42 | -1.19 | 1.02 |  | 2.66 (-2.76, 8.07) |
| Practice of healthy lifestyle | 34 | 42 | -3.87 | 3.11 |  | -3.23 (-9.28, 2.82) |
| **Shen, 2020 [47]** |  |  |  |  |  |  |
| BMI (kg/m^2^) | 245 | 298 | -0.3 | -0.3 |  | 0.03 (-0.12, 0.18) |
| BMI z score | 245 | 298 | -0.3 | -0.2 |  | 0.02 (-0.03, 0.07) |
| **Taghdisi, 2016 [48]** |  |  |  |  |  |  |
| Fruit intake | 94 | 90 | 0.60*** | 0.04 | 0.056 |  |
| Vegetable intake | 94 | 90 | 0.37*** | -0.01 | 0.38 |  |
| Fruit and vegetable intake | 94 | 90 | 0.51*** | 0.03 | 0.48 |  |
| **Physical activity** | | | | | | |
| **Andrade, 2014 & Ochoa-Avilés, 2017 [49, 50]** |  |  |  |  |  |  |
| BMI z score | 539 | 521 | -0.09 | -0.09 |  | -0.01 (-0.09, 0.06) |
| Overweight and obesity prevalence (%) | 539 | 521 | -1.62 | -1.62 |  | 0.02 (-0.05, 0.008) |
| Waist circumference (cm) | 543 | 530 | 2.2 | 2.9 |  | -0.84 (-1.68, -0.28)** |
| Fruit and vegetable intake | 546 | 532 | -54.0 | -38.4 |  | 23.88 (7.36, 40.40)** |
| Added sugar intake | 546 | 532 | -11.1 | -6.1 |  | -5.66 (-9.63, -1.65)** |
| Unhealthy snacking | 546 | 532 | -34.5 | 0.6 |  | -23.32 (-45.25, -1.37)* |
| Unhealthy snacking at school | 546 | 532 | 8.1 | 11.7 |  | -0.03 (-0.08, 0.06) |
| Breakfast intake | 546 | 532 | -4.4 | 5.2 |  | -0.03 (-0.06, 0.03) |
| Total fat | 546 | 532 | 0.4 | 0.9 |  | -0.45 (-1.20, 0.31) |
| **Gutiérrez-Martínez, 2018 [51]** |  |  |  |  |  |  |
| BMI z score | 44 | 42 | 0.05 | 0.1 |  |  |
| % of fat | 44 | 42 | 0.1 | 0.7 |  |  |
| **Leong, 2015 [52]** |  |  |  |  |  |  |
| Digit span test | NR | NR | 2.11* | -0.50 |  |  |
| **Telles, 2013 [53]** |  |  |  |  |  |  |
| BMI (kg/m^2^) | IG1: 49  IG2: 49 |  | IG1: 1.24***  IG2: 1.24*** |  |  |  |
| Word raw score | IG1: 43  IG2: 43 |  | IG1: 5.07**  IG2: 7.05*** |  |  |  |
| Color raw score | IG1: 43  IG2: 43 |  | IG1: 4.77**  IG2: 6.93*** |  |  |  |
| Color-word raw score | IG1: 43  IG2: 43 |  | IG1: 3.53**  IG2: 2.98** |  |  |  |
| **Guimarães, 2017 [54]** |  |  |  |  |  |  |
| Weight (kg) | 26 | 45 | -1.7 | 0.1 |  |  |
| % of fat mass | 26 | 45 | -0.4 | 0.1 |  |  |
| Fat-free mass | 26 | 45 | -1.3* | -0.3 |  |  |
| **Kargarfard, 2012 [55]** |  |  |  |  |  |  |
| BMI (kg/m^2^) | 206 | 60 | -0.2*** | -0.1* |  |  |
| **Li, 2014 [56]** |  |  |  |  |  |  |
| BMI (kg/m^2^) | 388 | 533 | -0.02 | 0.41 |  | -0.43 (-0.63, -0.23)*** |
| Overweight/obesity | 388 | 533 | -2.30 | 1.7 |  | 0.84 (0.46, 1.24) |
| Waist circumference (cm) | 386 | 532 | -1.06 | -0.70 |  | -0.38 (-0.81, 0.05) |
| Abdominal skinfold | 388 | 532 | -1.22 | -0.54 |  | -0.69 (-1.29, -0.10)* |
| **Zhang, 2019 [57]** |  |  |  |  |  |  |
| Achievement in the Chinese language | 236 | 224 |  |  |  | 0.61 (0.44, 0.78)*** |
| Achievement in the English language | 236 | 224 |  |  |  | 0.28 (0.09, 0.47)** |
| Achievement in maths | 236 | 224 |  |  |  | -0.27 (-0.42, -0.11) |
| **Nutrition subsidy** | | | | | | |
| **Chen, 2019 [58]** |  |  |  |  |  |  |
| Hemoglobin (g/dL) | IG1: 219  IG2: 210 | 439 | IG1: 0.4  IG2: -3.11 | 0.1 |  | IG1 vs C: 0.512  IG2 vs C: 4.490*** |
| Anemia | IG1: 219  IG2: 210 | 439 | IG1: 0.4  IG2: 0.7 | 0.1 |  | IG1 vs C: -0.005  IG2 vs C: -0.120*** |
| BMI z scores | IG1: 219  IG2: 210 | 439 | IG1: -0.01  IG2: 0.03 | -0.08 |  | IG1 vs C: 0.080  IG2 vs C: 0.123*** |
| Underweight | IG1: 219  IG2: 210 | 439 | IG1: 0.0  IG2: 0.0 | -0.3 |  | IG1 vs C: -0.032  IG2 vs C: -0.041 |
| Dietary diversity score | IG1: 219  IG2: 210 | 439 | IG1: 0.46  IG2: 0.67 | -0.51 |  | IG1 vs C: 0.956***  IG2 vs C: 1.263*** |
| **WASH intervention** | | | | | | |
| **Caruso, 2014 [59]** |  |  |  |  |  |  |
| School absence | IG1: 5490  IG2: 6772 | 5302 | IG1: 0.143  IG2: 0.140 | 0.143 |  | IG1 vs C: -0.003 (-0.031, 0.025)  IG2 vs C: 0.001 (-0.024, 0.026) |
| **Chard, 2019 [60]** |  |  |  |  |  |  |
| School absence | 6024 | 7147 |  |  |  | 1.01 (0.84, 1.20) |
| School enrollment | 6024 | 7147 |  |  |  | 1.07 (0.84, 1.37) |
| School drop-out | 6024 | 7147 |  |  |  | 0.56 (0.25, 1.25) |
| Grade progression | 6024 | 7147 |  |  |  | 1.07 (0.91, 1.25) |
|  |  |  |  |  |  |  |
| **Freeman, 2012 [61]** |  |  |  |  |  |  |
| School absence | IG1: 2015  IG2: 2008 | 2013 |  |  |  | IG1 vs C:  0.81 (0.49, 1.34)  IG2 vs C:  0.97 (0.55, 1.64) |
| **Theriault, 2014 [62]** |  |  |  |  |  |  |
| School absence | 517 | 571 | -1.5 | 0.8 | 0.8 |  |
| **Trinies, 2014 [63]** |  |  |  |  |  |  |
| Roll call absence | 4498 | 4444 |  |  |  | 1.23 (1.06, 1.42)** |
| 7-day absence recall | 4907 | 4823 |  |  |  | 0.93 (0.79, 1.09) |
| **Integrated multicomponent intervention** | | | | | | |
| **Barbosa Filho, 2019 [64]** |  |  |  |  |  |  |
| Fruit juice intake | 548 | 537 | 2.0 | -2.4 |  | 1.22 |
| Fruit intake | 548 | 537 | 0.2 | -0.6 |  | 0.96 |
| Vegetable intake | 548 | 537 | 0.9 | 1.1 |  | 0.91 |
| Soft drinks intake | 548 | 537 | 2.9 | 2.2 |  | 0.87 |
| Savory foods intake | 548 | 537 | -1.5 | 3.2 |  | 0.93 |
| Sweet intake | 548 | 537 | 8.2 | 7.4 |  | 1.63 |
| **da Costa, 2014 and de Sousa, 2014 [65, 66]** |  |  |  |  |  |  |
| Obesity | NR | NR | 0.5 | 0.9 |  |  |
| Waist circumference (cm) | NR | NR | 2.5** | 2.1** |  |  |
| Waist-to-height ratio | NR | NR | 0.9 | 1.6** |  |  |
| Fruit intake (daily) | 341 | 354 | 15.2 | 17.2 |  | 1.10 (0.92, 1.32) |
| Vegetable intake (daily) | 343 | 385 | -4.5 | -6.3 |  | 1.54 (1.19, 1.97) |
| Fruit intake (weekly) | 533 | 539 |  |  |  | 1.12 (0.94, 1.33) |
| Vegetable intake (weekly) | 260 | 259 |  |  |  | 1.21 (0.93, 1.56) |
| Dairy intake (weekly) | 649 | 581 |  |  |  | 0.79 (0.66, 0.95) |
| Beans intake (weekly) | 749 | 785 |  |  |  | 1.07 (0.84, 1.37) |
| Salty snacks intake (weekly) | 278 | 269 |  |  |  | 1.09 (0.84, 1.41) |
| Sweets intake (weekly) | 512 | 544 |  |  |  | 1.21 (1.02, 1.43) |
| Soft drinks intake (weekly) | 463 | 480 |  |  |  | 1.09 (0.92, 1.30) |
| **Erismann, 2017 [67]** |  |  |  |  |  |  |
| Total undernutrition | 176 | 184 | 2.8 | 3.3 |  | 0.9 (0.3, 3.9) |
| Stunting | 176 | 184 | 3.9 | 2.7 |  | 1.2 (0.6, 2.3) |
| Thinness | 176 | 184 | 2.3 | 1.6 |  | 1.1 (0.4, 2.8) |
| Underweight | 176 | 184 | -0.6 | -1.1 |  | NR |
| Overweight | 176 | 184 | -0.5 | 2.1 |  | 0 (0.0, 4.0) |
| Anemia | 176 | 184 | 5.1 | 10.4 |  | 0.7 (0.4, 1.5) |
| Height z score | NR | NR | NR | NR |  | 0.00 (-0.07, 0.08) |
| BMI z score | NR | NR | NR | NR |  | 0.05 (-0.08, 0.17) |
| Hemoglobin (g/dL) | NR | NR | NR | NR |  | -0.17 (-0.36, 0.02) |
| **Florence, 2020 [68]** |  |  |  |  |  |  |
| Waist circumference (cm) | 100 | 90 | 0.3 | 1.4 | 1.1* |  |
| BMI z score | 100 | 90 | -0.001 | -0.037 | 0.04*** |  |
| **Gall, 2018 and Müller, 2019 [69, 70]** |  |  |  |  |  |  |
| BMI z score | 264 | 255 |  |  |  | -0.17 (-0.24, -0.09)*** |
| Stunting (incidence) | 264 | 255 |  |  |  | 0.68 (0.23, 2.07) |
| Stunting (re-occurrence) | 264 | 255 |  |  |  | 0.68 (0.12, 3.96) |
| Stunting (prevalence) | 264 | 255 |  |  |  | 0.76 (0.09, 6.53) |
| Anemia (incidence) | 264 | 255 |  |  |  | 1.36 (0.53, 3.49) |
| Anemia (re-occurrence) | 264 | 255 |  |  |  | 0.82 (0.25, 2.62) |
| Anemia (prevalence) | 264 | 255 |  |  |  | 0.93 (0.38, 2.30) |
| Concentration performance | 265 | 398 |  |  |  | 2.93 (-5.01, 10.86) |
| Selective attention | 265 | 398 |  |  |  | -1.05 (-0.69, 2.78) |
| Academic performance | 265 | 398 |  |  |  | 0.34 (0.03, 0.65)* |
| **Jemmott III, 2011 and 2019 [71, 72]** |  |  |  |  |  |  |
| Fruit intake | 487 | 542 |  |  |  | 0.34 (0.05, 0.63)** |
| Vegetable intake | 487 | 542 |  |  |  | 0.47 (0.16, 0.78)** |
| Fried food intake | 487 | 542 |  |  |  | -0.22 (-0.36, -0.08)** |
| **Leme, 2016 and 2018 [73, 74]** |  |  |  |  |  |  |
| BMI (kg/m^2^) | 142 | 111 | 0.05 | 0.19 |  | -0.023 |
| BMI z score | 142 | 111 | -0.19 | -0.5* |  | -0.121 |
| Waist circumference (cm) | 142 | 111 | 2.12 | 3.84* |  | 0.102 |
| **Levy, 2012 [75]** |  |  |  |  |  |  |
| Change from normal to overweight | 498 | 499 |  |  |  | 0.89 (0.73, 1.11) |
| Change from overweight to obesity | 498 | 499 |  |  |  | 0.68 (0.51, 0.91)** |
| **Morales-Ruán, 2014 [76]** |  |  |  |  |  |  |
| Prevalence of overweight and obesity | 510 | 509 |  |  |  | 0.46 (0.27, 0.81)** |
| **Saraf, 2015 [77]** |  |  |  |  |  |  |
| Fruit intake | 1014 | 1060 | 14.2* | 4.2* | 10.0** |  |
| Vegetable intake | 1014 | 1060 | 4.65* | -2.7 | 7.2** |  |
| Fried foods intake | 1014 | 1060 | -6.1* | -0.1 | 6.0* |  |
| Salted snacks intake | 1014 | 1060 | 7.1 | 3.6 | 3.5 |  |
| **Shirazi, 2019 [78]** |  |  |  |  |  |  |
| Breakfast practice | 115 | 115 | 2.3 | 0.0 | 2.3*** |  |
| Fruit intake | 115 | 115 | 1.6 | 0.1 | 1.5*** |  |
| Vegetable intake | 115 | 115 | 1.1 | 0.0 | 1.1*** |  |
| Snacks intake | 115 | 115 | 1.5 | 0.0 | 1.5*** |  |
| Fast food intake | 115 | 115 | 1.7 | -0.1 | 1.8*** |  |
| **Shrestha, 2020 [79]** |  |  |  |  |  |  |
| Prevalence of stunting | IG1: 172  IG2: 197 | 313 | IG1: 1.8  IG2: -1.6 | 0.8 |  | IG1 vs C:  1.17 (0.62, 0.54)  IG2 vs C:  0.88 (0.49, 1.56) |
| Prevalence of thinness | IG1: 172  IG2: 197 | 313 | IG1: 0.7  IG2: 4.2 | -5.2 |  | IG1 vs C:  1.09 (0.48, 2.48)  IG2 vs C:  2.10 (0.88, 5.02)** |
| Prevalence of anemia | IG1: 172  IG2: 197 | 313 | IG1: 23.2  IG2: -1.0 | 18.6 |  | IG1 vs C:  3.77 (2.17, 6.56)  IG2 V C:  0.94 (0.59, 1.51)** |
| **Singhal, 2010 [80]** |  |  |  |  |  |  |
| Height (cm) | 39 | 41 | 0.76*** | 1.02*** |  |  |
| Weight (kg) | 39 | 41 | 1.52 | 0.69*** |  |  |
| BMI (kg/m^2^) | 39 | 41 | -0.07 | -0.06 |  |  |
| Waist circumference (cm) | 39 | 41 | -0.65 | 0.65 |  |  |
| Waist-to-hip ratio | 39 | 41 | -0.008* | 0.005 |  |  |
| Waist-to-height ratio | 39 | 41 | -0.005* | 0.001 |  |  |
| **Styen, 2015 [81]** |  |  |  |  |  |  |
| Dietary diversity score | 424 | 360 | 0.39* | 0.35 | 0.04 |  |
| Fat intake score | 424 | 360 | 0.36* | 0.38* | -0.03 |  |
| Sugar intake score | 424 | 360 | 0.11 | 0.38 | -0.27 |  |
| **Thakur, 2016 [82]** |  |  |  |  |  |  |
| Height (meters) | 157 | 216 | 0.03 | 0.02 |  | -0.04 (-0.14, 0.05) |
| Weight (kg) | 157 | 216 | 4.39 | 2.71 |  | -0.08 (-0.15, 0.00)* |
| BMI (kg/m^2^) | 157 | 216 | 0.88 | 0.47 |  | -0.09 (-0.19, 0.01) |
| Waist circumference | 157 | 216 | 5.78 | 4.21 |  | -0.14 (-0.25, -0.03)** |
| Hip circumference | 157 | 216 | 3.28 | 3.0 |  | -0.1 (-0.11, 0.09) |
| Waist hip ratio | 157 | 216 | 0.04 | 0.03 |  | -0.10 (-0.29, 0.08) |
| **Wang, 2018 [83]** |  |  |  |  |  |  |
| BMI (kg/m^2^) | 5275 | 4583 | 0.22 | 0.46 |  | -0.3 (-0.5, -0.1)** |
| BMI z score | 5275 | 4583 | 0.07 | 0.16 |  | -0.1 (-0.2, -0.03)** |
| % obese | 5275 | 4583 | 0.6 | 2.3 |  | 0.7 (0.6, 0.9)** |
| % overweight or obese | 5275 | 4583 | 0.9 | 2.9 |  | 0.8 (0.7, 1.0)* |
| **Xu, 2015 [84]** |  |  |  |  |  |  |
| BMI (kg/m^2^) | 605 | 503 | -0.32 | -0.29 | -0.03 | NR |
| Red meat consumption | 605 | 503 |  |  |  | 1.50 (1.15, 1.95)* |
| Fried snacks consumption | 605 | 503 |  |  |  | 1.08 (0.81, 1.44) |
| Soft drinks consumption | 605 | 503 |  |  |  | 0.89 (0.67, 1.19) |
| Vegetable intake | 605 | 503 |  |  |  | 1.20 (0.92, 1.55) |
| **Xu, 2017 [85]** |  |  |  |  |  |  |
| BMI (kg/m^2^) | 3476 | 3398 | 0.6* | 0.8* |  | -0.3 (-0.4, -0.2)*** |
| BMI z score | 3476 | 3398 | -0.11* | 0.03* |  | -0.15 (-0.18, -0.11)*** |
| % body fat | 3476 | 3398 | 1.1* | 1.8* |  | -0.8 (-0.9, -0.6)*** |
| Waist circumference (cm) | 3476 | 3398 | 3.1* | 3.6* |  | -0.5 (-0.6, -0.3)*** |
| Overweight | 3476 | 3398 | -1.2 | 0.2 |  | 0.9 (0.8, 1.1) |
| Obese | 3476 | 3398 | 1.4* | 1.3* |  | 1.0 (0.9, 1.1) |
| Overweight and obese | 3476 | 3398 | 0.2 | 1.5* |  | 0.9 (0.7, 1.0) |
| **Alaofè, 2009 [86]** |  |  |  |  |  |  |
| Anemia (%) | 34 | 34 | 32 | 85 | 53* |  |
| Iron deficiency anemia (%) | 34 | 34 | 26 | 56 | 30* |  |
| BMI (kg/m^2^) |  |  |  |  |  | No significant differences between IG and C; data not reported |
| **Tamiru, 2016a and 2016b [87, 88]** |  |  |  |  |  |  |
| Height for age | 500 | 500 |  |  |  | 1.65 (0.90, 3.07) |
| Consumption of animal source food | 500 | 500 |  |  |  | 0.26 (0.16, 0.42)* |
| Dietary diversity score | 500 | 500 |  |  |  | 2.55 (1.55, 3.50)*** |
| **Wei, 2019 [89]** |  |  |  |  |  |  |
| Weight (kg) | 181 | 186 |  |  |  | 0.17 (-1.11, 1.44) |
| Thinness | 181 | 186 |  |  |  | 1.03 (0.27, 3.94) |
| Overweight | 181 | 186 |  |  |  | 0.54 (0.23, 1.30) |
| Stunting | 181 | 186 |  |  |  | 0.48 (0.26, 0.87)* |
| Health knowledge | 181 | 186 |  |  |  | 0.53 (0.24, 0.81)*** |
| School absence | 181 | 186 |  |  |  | 0.89 (0.60, 1.33) |

**Online Additional File 1:** PRISMA checklist 2009.

| **Section/topic** | **#** | **Checklist item** | **Reported on page #** |  |
| --- | --- | --- | --- | --- |
| **TITLE** | | | |  |
| Title | 1 | Identify the report as a systematic review, meta-analysis, or both. | 1 |  |
| **ABSTRACT** | | | |  |
| Structured summary | 2 | Provide a structured summary including, as applicable: background; objectives; data sources; study eligibility criteria, participants, and interventions; study appraisal and synthesis methods; results; limitations; conclusions and implications of key findings; systematic review registration number. | 2-3 |  |
| **INTRODUCTION** | | | |  |
| Rationale | 3 | Describe the rationale for the review in the context of what is already known. | 4-5 |  |
| Objectives | 4 | Provide an explicit statement of questions being addressed with reference to participants, interventions, comparisons, outcomes, and study design (PICOS). | 5 |  |
| **METHODS** | | | |  |
| Protocol and registration | 5 | Indicate if a review protocol exists, if and where it can be accessed (e.g., Web address), and, if available, provide registration information including registration number. | 5 |  |
| Eligibility criteria | 6 | Specify study characteristics (e.g., PICOS, length of follow-up) and report characteristics (e.g., years considered, language, publication status) used as criteria for eligibility, giving rationale. | 5-6 |  |
| Information sources | 7 | Describe all information sources (e.g., databases with dates of coverage, contact with study authors to identify additional studies) in the search and date last searched. | 6 |  |
| Search | 8 | Present full electronic search strategy for at least one database, including any limits used, such that it could be repeated. | Additional File 2 |  |
| Study selection | 9 | State the process for selecting studies (i.e., screening, eligibility, included in systematic review, and, if applicable, included in the meta-analysis). | 6-7 |  |
| Data collection process | 10 | Describe method of data extraction from reports (e.g., piloted forms, independently, in duplicate) and any processes for obtaining and confirming data from investigators. | 7 |  |
| Data items | 11 | List and define all variables for which data were sought (e.g., PICOS, funding sources) and any assumptions and simplifications made. | 7 |  |
| Risk of bias in individual studies | 12 | Describe methods used for assessing risk of bias of individual studies (including specification of whether this was done at the study or outcome level), and how this information is to be used in any data synthesis. | 7-8 |  |
| Summary measures | 13 | State the principal summary measures (e.g., risk ratio, difference in means). | 9 |  |
| Synthesis of results | 14 | Describe the methods of handling data and combining results of studies, if done, including measures of consistency (e.g., I^2^) for each meta-analysis. | Not applicable |  |
| Risk of bias across studies | 15 | Specify any assessment of risk of bias that may affect the cumulative evidence (e.g., publication bias, selective reporting within studies). | 7-8 |  |
| Additional analyses | 16 | Describe methods of additional analyses (e.g., sensitivity or subgroup analyses, meta-regression), if done, indicating which were pre-specified. | Not applicable |  |
| **RESULTS** | | | |  |
| Study selection | 17 | Give numbers of studies screened, assessed for eligibility, and included in the review, with reasons for exclusions at each stage, ideally with a flow diagram. | 9 and Figure 1 |  |
| Study characteristics | 18 | For each study, present characteristics for which data were extracted (e.g., study size, PICOS, follow-up period) and provide the citations. | Table 1 |  |
| Risk of bias within studies | 19 | Present data on risk of bias of each study and, if available, any outcome level assessment (see item 12). | Supplementary Table 1 |  |
| Results of individual studies | 20 | For all outcomes considered (benefits or harms), present, for each study: (a) simple summary data for each intervention group (b) effect estimates and confidence intervals, ideally with a forest plot. | Supplementary Table 2 |  |
| Synthesis of results | 21 | Present results of each meta-analysis done, including confidence intervals and measures of consistency. | 9-20 |  |
| Risk of bias across studies | 22 | Present results of any assessment of risk of bias across studies (see Item 15). | Table S2 |  |
| Additional analysis | 23 | Give results of additional analyses, if done (e.g., sensitivity or subgroup analyses, meta-regression [see Item 16]). | Not applicable |  |
| **DISCUSSION** | | | |  |
| Summary of evidence | 24 | Summarize the main findings including the strength of evidence for each main outcome; consider their relevance to key groups (e.g., healthcare providers, users, and policy makers). | 20-21 |  |
| Limitations | 25 | Discuss limitations at study and outcome level (e.g., risk of bias), and at review-level (e.g., incomplete retrieval of identified research, reporting bias). | 24 |  |
| Conclusions | 26 | Provide a general interpretation of the results in the context of other evidence, and implications for future research. | 25 |  |
| **FUNDING** | | | |  |
| Funding | 27 | Describe sources of funding for the systematic review and other support (e.g., supply of data); role of funders for the systematic review. | 26 |  |

**Online Additional File 2:** PubMed search strategy.

| **No.** | **Concept** | **PubMed search terms** |
| --- | --- | --- |
| #1 | **Randomized controlled trial**  **Controlled before-after studies**  **Quasi-experimental studies** | ("randomized controlled trial"[pt] OR "controlled clinical trial"[pt] OR "clinical trials as topic"[mesh] OR "random allocation"[mesh] OR "double-blind method"[mesh] OR "single-blind method"[mesh] OR "clinical trial"[pt] OR "research design"[mesh:noexp] OR "comparative study"[pt] OR "evaluation studies"[pt] OR "follow-up studies"[mesh] OR "prospective studies"[mesh] OR "cross-over studies"[mesh] OR "Controlled Before-After Studies"[Mesh] OR "clinical trial"[tw] OR ((singl*[tw] OR doubl*[tw] OR trebl*[tw]) AND (mask*[tw] OR blind*[tw])) OR placebo*[tw] OR quasi experiment*[tiab] OR quasiexperiment*[tiab] OR random*[tw] OR "control"[tw] OR "controls"[tw] OR prospectiv*[tw] OR volunteer*[tw]) |
| #2 | **Nutrition and health interventions**  (Dietary supplements, healthy diet, healthy eating, healthy nutrition, overweight, anemia, obesity, weight control, weight management, micronutrient supplementation diet/nutrition education, school meal, physical activity, school garden, and WASH, and nutrition policy) | **("Health Education"[Mesh:NoExp] OR "Health Promotion"[Mesh] OR "Adolescent Health Services"[Mesh] OR "Preventive Health Services"[Mesh:NoExp] OR preventive health[tiab] OR ("Dietary Supplements"[Mesh] OR "Diet, Healthy"[Mesh] OR "Fruit"[Mesh:NoExp] OR "Vegetables"[Mesh] OR "Meals"[Mesh] OR "Anemia"[Mesh] OR "Overweight"[Mesh] OR anemia[tiab] OR anaemia[tiab] OR healthy diet*[tiab] OR healthy eating[tiab] OR healthy food*[tiab] OR healthy nutrition*[tiab] OR nutrition counsel*[tiab] OR nutritional counsel*[tiab] OR obesity[tiab] OR over weight[tiab] OR overweight[tiab] OR weight control[tiab] OR weight management[tiab] OR "Malnutrition"[Mesh] OR "malnutrition"[tiab] OR "undernutrition"[tiab] OR "undernutritional"[tiab] OR "undernourished"[tiab] OR "undernourishment"[tiab] OR "wasting"[tiab] OR "wasted"[tiab] OR "stunting"[tiab] OR "stunted"[tiab] OR "thinness"[MeSH] OR "thinness"[tiab] OR "underweight"[tiab] OR "underweights"[tiab] OR breakfast*[tiab] OR contraception education*[tiab] OR contraceptive education*[tiab] OR depressive[tiab] OR diet education[tiab] OR dietary education[tiab] OR dietary intervention[tiab] OR dietary supplement*[tiab] OR folic acid supplement*[tiab] OR fruit[tiab] OR fruits[tiab] OR health education[tiab] OR health intervention[tiab] OR health promotion[tiab] OR healthy eating[tiab] OR healthy food*[tiab] OR healthy diet*[tiab] OR lunch*[tiab] OR meals[tiab] OR micronutrient supplement*[tiab] OR nutrient supplement*[tiab] OR nutrition education[tiab] OR nutrition intervention[tiab] OR nutritional education[tiab] OR nutritional intervention[tiab] OR nutritional supplement*[tiab] OR school meal*[tiab] OR snack*[tiab] OR vegetable*[tiab] OR vitamin supplement*[tiab] OR "exercise"[MeSH] OR "exercise" [tiab] OR ("physical"[tiab] AND "activity"[tiab]) OR "physical activity"[tiab] OR garden*[tiab] OR "WASH" [tiab]) OR "hygiene"[MeSH] OR "hygiene"[tiab] OR "sanitation"[MeSH] OR "sanitation"[tiab] OR "nutrition policy"[MeSH] OR ("nutrition"[tiab] AND "policy"[tiab]) OR "nutrition policy"[tiab]) AND ("School Health Services"[Mesh] OR "Schools"[Mesh:NoExp] OR government school*[tiab] OR high school*[tiab] OR highschool*[tiab] OR junior high[tiab] OR middle school*[tiab] OR private school*[tiab] OR public school*[tiab] OR school based[tiab] OR school breakfast*[tiab] OR school day[tiab] OR school health[tiab] OR school lunch*[tiab] OR school meal*[tiab] OR school nutrition*[tiab] OR school setting[tiab] OR schoolday[tiab] OR secondary school*[tiab])** |
| #3 | **Adolescents** | ("Adolescent"[Mesh] OR adolescent[tiab] OR adolescents[tiab] OR adolescence[tiab] OR teen[tiab] OR teens[tiab] OR teenage*[tiab] OR high school*[tiab] OR highschool*[tiab] OR middle school*[tiab] OR junior high*[tiab] OR preadolescen*[tiab] OR prepupert*[tiab] OR pubert*[tiab] OR secondary school*[tiab] OR pubescen*[tiab] OR youth[tiab] OR youths[tiab] OR young people[tiab] OR 10 years old[tiab] OR 11 years old[tiab] OR 12 years old[tiab] OR 13 years old[tiab] OR 10 years of age[tiab] OR 11 years of age[tiab] OR 12 years of age[tiab] OR 13 years of age[tiab] OR 3rd grade*[tiab] OR 4th grade*[tiab] OR 5th grade*[tiab] OR 6th grade*[tiab] OR age 10[tiab] OR age 11[tiab] OR age 12[tiab] OR age 13[tiab] OR aged 10[tiab] OR aged 11[tiab] OR aged 12[tiab] OR aged 13[tiab] OR age ten[tiab] OR age eleven[tiab] OR age twelve[tiab] OR age thirteen[tiab] OR grade 3[tiab] OR grade 4[tiab] OR grade 5[tiab] OR grade 6[tiab] OR grades 3[tiab] OR grades 4[tiab] OR grades 5[tiab] OR grades 6[tiab] OR third grade*[tiab] OR fourth grade*[tiab] OR fifth grade*[tiab] OR sixth grade*[tiab]) |
| #4 | **Low- and middle-income countries** | ("Developing Countries"[Mesh] OR developing countr*[tiab] OR under developed countr*[tiab] OR lmic*[tiab] OR ((less developed[tiab] OR low income[tiab] OR lower income[tiab] OR low and middle income[tiab] OR low middle income[tiab] OR resource poor[tiab] OR resource constrained[tiab] OR low resource[tiab] OR limited resource*[tiab] OR resource limited[tiab]) AND (country[tiab] OR countries[tiab] OR region[tiab] OR regions[tiab] OR setting*[tiab] OR area[tiab] OR areas[tiab])) OR "Africa South of the Sahara"[Mesh] OR "Central America"[Mesh] OR "South America"[Mesh] OR "Latin America"[Mesh] OR "Caribbean Region"[Mesh] OR "Mexico"[Mesh] OR "Asia"[Mesh:NoExp] OR "Asia, Central"[Mesh] OR "Asia, Northern"[Mesh] OR "Asia, Southeastern"[Mesh] OR "Asia, Western"[Mesh] OR "China"[Mesh] OR "Korea"[Mesh] OR "Mongolia"[Mesh] OR Afghan*[tiab] OR Africa[tiab] African[tiab] OR Algeria*[tiab] OR American Samoa*[tiab] OR Angola*[tiab] OR Argentin*[tiab] OR Bangladesh*[tiab] OR Barbad*[tiab] OR Belorussian[tiab] OR Beliz*[tiab] OR Benin*[tiab] OR Bhutan*[tiab] OR Bolivia*[tiab] OR Botswan*[tiab] OR Brazil*[tiab] OR "Burkina Faso"[tiab] OR Burkinabe[tiab] OR Burund*[tiab] OR Cambodia*[tiab] OR Cameroon*[tiab] OR "Cape Verde"[tiab] OR "Cape Verdean"[tiab] OR "Central African Republic"[tiab] OR Chad*[tiab] OR Chile*[tiab] OR China[tiab] OR Chinese[tiab] OR Colombia*[tiab] OR Comoros[tiab] OR Comorian[tiab] OR Congo[tiab] OR Congolese[tiab] OR Costa Rica*[tiab] OR "Côte d’Ivoire"[tiab] OR “Ivory Coast”[tiab] OR Ivorian[tiab] OR Croatia*[tiab] OR Croat[tiab] OR Cuba*[tiab] OR Djibouti*[tiab] OR Dominica*[tiab] OR "Dominican Republic"[tiab] OR Ecuador*[tiab] OR Egypt*[tiab] OR "El Salvador"[tiab] OR Salvadorian[tiab] OR "Equatorial Guinea"[tiab] OR Guinean[tiab] OR Eritrea*[tiab] OR Ethiopia*[tiab] OR Fiji*[tiab] OR Gabon*[tiab] OR Gambia*[tiab] OR Gaza[tiab] OR Gazan[tiab] OR Ghana[tiab] OR Ghanaian[tiab] OR Grenad*[tiab] OR Guatemala*[tiab] OR Guinea[tiab] OR Guyan*[tiab] OR Haiti*[tiab] OR Hondura*[tiab] OR Hungar*[tiab] OR India[tiab] OR Indian[tiab] OR Indonesia*[tiab] OR Iran*[tiab] OR Iraq*[tiab] OR Jamaica*[tiab] OR Jordan*[tiab] OR Kenya[tiab] OR Kenyan[tiab] OR Kiribati[tiab] OR Korea*[tiab] OR Kyrgy*[tiab] OR Laos[tiab] OR Laotian*[tiab] OR Lebanon[tiab] OR Lebanese[tiab] OR Lesotho[tiab] OR Liberia*[tiab] OR Libya*[tiab] OR Macedonia*[tiab] OR Madagasca*[tiab] OR Malawi*[tiab] OR Malaysia*[tiab] OR Maldives[tiab] OR Maldivian[tiab] OR Mali[tiab] OR Malian*[tiab] OR "Marshall Islands"[tiab] OR Mauritania*[tiab] OR Mauritius[tiab] OR Mauritian[tiab] OR Mayotte[tiab] OR Mexic*[tiab] OR Micronesia*[tiab] OR Moldov*[tiab] OR Mongolia*[tiab] OR Morocc*[tiab] OR Mozambique[tiab] OR Mozambican[tiab] OR Myanmar[tiab] OR Namibia*[tiab] OR Nepal*[tiab] OR Nevis[tiab] OR Nicaragua*[tiab] OR Niger*[tiab] OR "Northern Mariana Islands"[tiab] OR Oman*[tiab] OR Pakistan*[tiab] OR Palau*[tiab] OR Panama*[tiab] OR "Papua New Guinea"[tiab] OR Paraguay*[tiab] OR Peru*[tiab] OR Philippine*[tiab] OR Filipino*[tiab] OR Poland[tiab] OR Polish[tiab] OR Rwanda*[tiab] OR Samoa*[tiab] OR Sao Tome*[tiab] OR Principe[tiab] OR Senegal*[tiab] OR Seychell*[tiab] OR Sierra Leon*[tiab] OR Solomon Island*[tiab] OR Somali*[tiab] OR South Africa*[tiab] OR Sri Lanka*[tiab] OR "Saint Kitts"[tiab] OR "St Kitts"[tiab] OR "Saint Lucia"[tiab] OR "St Lucia"[tiab] OR "Saint Vincent" [tiab] OR "St Vincent"[tiab] OR Sudan*[tiab] OR Suriname*[tiab] OR Swaziland Or Swazi[tiab] OR Syria*[tiab] OR Tajik*[tiab] OR Tanzania*[tiab] OR Thailand[tiab] OR Thai[tiab] OR "Timor Leste"[tiab] OR Togo*[tiab] OR Tonga*[tiab] OR Tunisia*[tiab] OR Turky[tiab] OR Turkish[tiab] OR Uganda*[tiab] OR Uruguay*[tiab] OR Vanuat*[tiab] OR Venezuela*[tiab] OR Vietnam*[tiab] OR "West Bank"[tiab] OR Yemen*[tiab] OR Zambia*[tiab] OR Zimbabwe*) |
